# Thermal diffusivity microscope: Zooming in on anisotropic heat transport

**DOI:** 10.1126/sciadv.ads6538

**Published:** 2025-02-26

**Authors:** Neetu Lamba, Braulio Beltrán-Pitarch, Tianbo Yu, Muhamed Dawod, Alex Berner, Benny Guralnik, Andrey Orekhov, Nicolas Gauquelin, Yaron Amouyal, Johan Verbeeck, Ole Hansen, Nini Pryds, Dirch Hjorth Petersen

**Affiliations:** ^1^ Department of Energy Conversion and Storage, Technical University of Denmark (DTU), Building 310, DK-2800 Kgs. Lyngby, Denmark.; ^2^ KLA, Diplomvej 373B, DK-2800 Kgs. Lyngby, Denmark.; ^3^ Department of Civil and Mechanical Engineering, Technical University of Denmark, DK-2800 Kgs. Lyngby, Denmark.; ^4^ Department of Materials Science and Engineering, Technion–Israel Institute of Technology, Haifa 32000, Israel.; ^5^ EMAT, University of Antwerp, 2020 Antwerp, Belgium.; ^6^ DTU Nanolab–National Centre for Nano Fabrication and Characterization, Technical University of Denmark, Ørsteds Plads 347, DK-2800 Kgs. Lyngby, Denmark.

## Abstract

Anisotropic heat–conducting materials play crucial roles in designing electronic, optoelectronic, and thermoelectric devices, where temperature and thermal stress are important. Despite substantial research efforts, a major obstacle to determining the anisotropic thermal diffusivity tensor in polycrystalline systems is the need for a robust, direct, and nondestructive technique to distinguish between distinct thermal diffusivities. Here, we demonstrate a conceptually unique thermal diffusivity microscope capable of performing high-resolution local measurements of anisotropic thermal diffusivity. The microscope features a unique micro four-point probe for fast, nondestructive scanning without calibration or extra sample preparation. It measures anisotropic thermal diffusivity based on thermal delay from a single heater. Through a series of experiments, we demonstrate that the anisotropy of the measured thermal diffusivity correlates excellently with the crystallographic direction of prototypical Bi_2_Te_3_. The anisotropic heat transport shows that the lattice contribution dominates the heat transport for both in- and out-of-plane directions.

## Teaser

A calibration- and preparation-free method for high-resolution anisotropic thermal diffusivity mapping is presented.

## INTRODUCTION

Thermal diffusivity is crucial in various fields of science and engineering as it governs heat transport within materials, e.g., for thermoelectric materials (*1*–*6*), thermal barrier coatings (*7*–*9*), high-power devices (*10*, *11*), and microelectronics (*12*, *13*). In many cases, materials exhibit a uniform behavior in conducting heat, meaning that their thermal diffusivity remains the same regardless of the direction of heat flow. However, there are numerous instances where this uniformity does not hold. Anisotropic thermal diffusivity arises when a material exhibits directional dependence in its ability to conduct heat, e.g., along different crystallographic orientations (*14*–*17*). The directional dependency in a material is linked to the phonon and electron transport, which is described by the phonon and electron dispersion relations, atomic mass, bond strength, charge concentration, and mobility. Other factors, like crystal grain size and shape and specific conditions at grain boundaries (GBs), may also cause anisotropic transport (*18*–*20*).

Extensive efforts have been made to characterize heat transport in bulk materials. The measured quantity is either thermal diffusivity, *D*, or thermal conductivity, κ = ρ*c*_*p*_*D*, where ρ is the mass density and *c*_*p*_ the specific heat capacity. To date, a variety of measurement techniques are available for the characterization of isotropic heat transport in both bulk and thin film materials within a broad temperature range. Local thermal diffusivity measurements are much more challenging than the characterization of electrical properties routinely measured locally (*21*, *22*) due to notable uncertainties related to heat transport. Most heat transport measurements are limited to isotropic materials, whereas the measurement of anisotropic heat transport requires one of the principal diffusivities to be aligned with the measurement direction. This restriction poses a challenge in accurately assessing the thermal properties of materials with more complex or undefined orientations. To probe the thermal diffusivity tensor in anisotropic materials, the currently available techniques are as follows: (i) laser flash analysis (LFA) (*23*), which is a transient thermal measurement technique where the diffusivity tensor can then be determined by evaluating a single crystal sample that has been sliced in various orientations (*5*, *24*). By combining LFA with electron backscatter diffraction (EBSD) or orientation imaging microscopy (*25*, *26*), it is possible to assign an average thermal diffusivity along different crystallographic directions in a polycrystalline material (*27*). (ii) The 3ω method (*28*), is a highly effective method in detecting minute variations in materials with low conductivities. The 3ω method has also been considered for obtaining the anisotropic thermal conductivity tensor (*28*). This technique’s complex setup and calibration, alongside the need for in-depth heat transfer knowledge and intricate mathematical models, make it challenging. The demanding sample preparation and specific requirements, like a thin metal line heater, render the 3ω method less ideal for routine measurements. (iii) Time-domain thermoreflectance (TDTR) (*29*–*31*), spatially resolved frequency domain thermoreflectance (FDTR) (*32*), and spatial domain thermoreflectance (*30*, *33*) are different variants of transient thermal measurement techniques for thin films or small samples. Using these transient methods, in combination with EBSD, it is possible to analyze thermal diffusivity values in specific directions (*22*, *34*). However, the accuracy of the extracted properties depends on the accuracy of the chosen model and assumptions made during the fitting process. Time-resolved magneto-optic Kerr effect thermometry is another technique with the potential to measure anisotropic thermal conductivity (*35*–*37*). In addition, these methods are not calibration free and require depositing additional metal (e.g., Au) layers on the surface. The lateral resolution of these techniques is typically on the order of several micrometers (*30*, *33*). (iv) Transient absorption microscopy (TAM) (*38*) is a technique used to study ultrafast dynamics in materials, including thermal transport processes. The TAM is sensitive to thermal transport near the surface with a lateral spatial resolution of about 100 nm (*39*). TAM, TDTR, and FDTR involve complex data analysis techniques to extract thermal transport information and require the deposition of a thin metal layer (10 to 20 nm), which may further complicate the analysis of anisotropic heat transport.

Despite ongoing advancements in experimental methods for estimating the anisotropic thermal diffusivity tensor, a direct, fast, calibration- and preparation-free, and highly resolved determination of anisotropic heat transport across varying crystallographic orientations remains challenging. Micro four-point probe (M4PP) is a commercially established method for advanced characterization (*40*–*42*), such as measuring sheet resistance, carrier density, and electron mobility. Recently, we demonstrated the possibility of estimating the isotropic thermal diffusivity from frequency-dependent M4PP measurements (*43*). However, this method depends on the isotropic thermal diffusivity, which necessitates fitting the measured second harmonic phase delay (from two heaters) with frequency. This method also requires probe calibration, assumptions about the electrodes contact geometries, and a heat transport model limited to isotropic materials. In addition, the potential contribution from the cold finger effect (parasitic heat transfer down the four-point probe electrodes) was also neglected.

Here, we propose an innovative methodology to accurately map anisotropic thermal diffusivity with remarkable precision, using a fast, nondestructive, direct, and calibration-free technique with no additional sample preparation. The proposed method is based on measuring thermal delay from a single heater. The method proposed here does not require a variation of frequency, and it is free from errors due to the cold finger effect, contact geometries, and probe calibration, emphasizing notable knowledge enhancement in this area. The method is demonstrated on two well-studied thermoelectric materials, Bi_2_Te_3_ and Sb_2_Te_3_, known as the best thermoelectric materials for various applications of energy conversion and cooling, and offers the possibility to tailor physical properties via GB engineering, topological insulation, energy filtering, and more (*5*, *27*, *44*–*48*). We investigate the correlation between local variation in anisotropic thermal diffusivity and the underlying microstructure using EBSD and high-angle annular dark field–scanning transmission electron microscopy (HAADF-STEM). Our findings show qualitatively good agreement with the scarce values reported in the literature. This technique is of both fundamental and practical importance for developing strategies to design more efficient heat transfer pathways in conducting materials. To determine the thermal diffusivity for different crystallographic orientations, we measure thermal phase delay at a constant frequency due to a single heater, as described in detail in Materials and Methods and the Supplementary Materials (Theoretical background).

## RESULTS

**Fig. 1.**
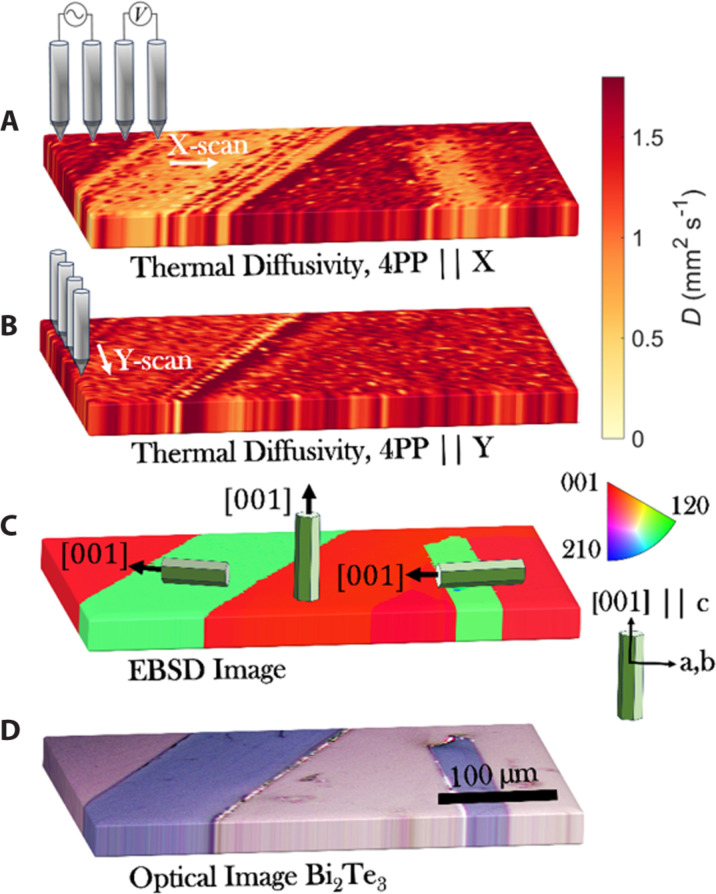
High-resolution thermal diffusivity area maps of Bi_2_Te_3_. Illustration of the results using the M4PP setup to map the thermal diffusivity of the Bi_2_Te_3_ grains of different orientations. (**A**) The measured thermal diffusivity using the M4PP in X-scan direction and (**B**) the measured thermal diffusivity using the M4PP in Y-scan direction. The thermal diffusivity values obtained in the two different scan directions are encoded in the color code to the right. The upper limit of the color bar corresponds to the cutoff at 1.8 mm^2^/s. (**C**) EBSD images of the grains, in IPF map notation and an eye guide arrow indicating the normal [001] of the crystal structure. (**D**) is an optical image of the Bi_2_Te_3_ grains.

Figure 1 displays the M4PP (fig. S3 shows an image of the M4PP during measurement) randomly mapped 300 μm–by–400 μm region with step size of 5 μm, illustrating the local thermal diffusivity across different grains for Bi_2_Te_3_ (see Materials and Methods and Supplementary Materials and Methods for more information on the preparation of the materials). Similar mappings of Sb_2_Te_3_ are shown in the Supplementary Materials (fig. S15). Bi_2_Te_3_ and Sb_2_Te_3_ are two materials that have a distinctive rhombohedral layered crystal structure stacked by weak van der Waals forces along the *c* axis. Within the layers, atoms are bonded by strong covalent bonds (see fig. S4). Such layered structure results in highly anisotropic transport properties.

**Fig. 2.**
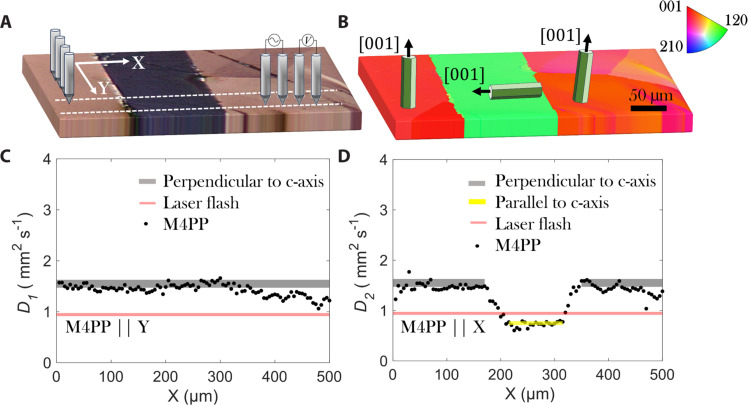
Line scans of thermal diffusivity. Thermal diffusivity imaging across individual GBs in large-grained Bi_2_Te_3_. (**A**) Optical micrograph showing the grains as well the orientation of probes, i.e., perpendicular (M4PP||X) and parallel (M4PP||Y) to the GBs. (**B**) EBSD and the IPF notation with the different grain orientation for the Bi_2_Te_3_ sample investigated in this work. (**C**) Thermal diffusivity line-scan (M4PP||Y) values (black dots) obtained using the M4PP technique along the white dashed line marked in (A). For comparison, we have included the gray “band” indicating the perpendicular to *c* axis values from the literature (*49*, *51*) and the red-dash line showing the value measured using LFA. (**D**) Thermal diffusivity line-scan (M4PP||X) values (black dots) obtained using the M4PP technique along the line marked in (A). For comparison, we have included the gray band indicating the perpendicular to *c* axis values from the literature, the parallel to *c* axis values in yellow taken from the literature, and the red-dash line showing the value measured using LFA. The thermal diffusivity lines are taken across a line of 500 μm in length, with a scanning step size of 5 μm. A clear change in the thermal diffusivity was detected depending on the orientation of the grains.

**Fig. 3.**
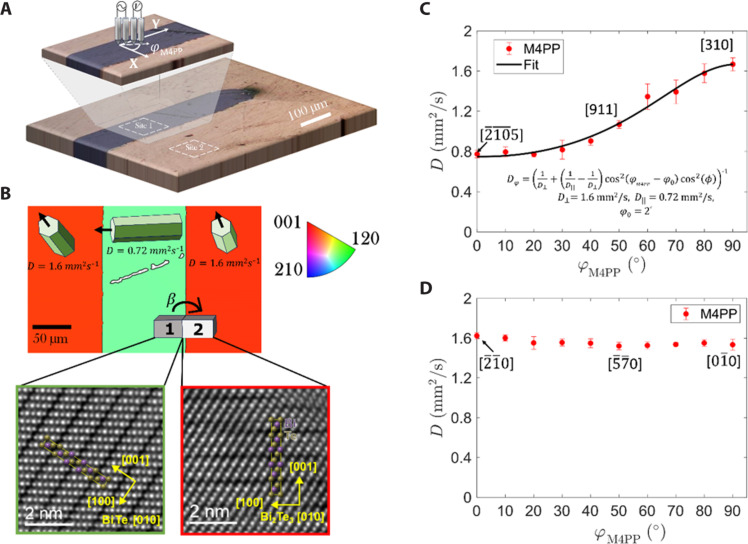
Crystallographic angle scans of thermal diffusivity. (**A**) Polarized optical image depicting scan sites 1 and 2 and a zoom-in image of site 1. A schematic illustration of the M4PP geometry and the angle dependence scan of sites 1 and 2 for 10 different angles is also shown. (**B**) The top image shows an EBSD scan with grain colors corresponding to IPF||Z notation, indicating grain orientation for the studied region of the specimen. Blocks 1 and 2 are the side view of the FIB lamella where HAADF-STEM imaging was performed. The bottom left and right images correspond to the atomic resolution HAADF-STEM images acquired on the two grains of the prepared lamella from blocks 1 and 2, respectively, clearly showing the van der Waals gaps (dark contrast) parallel to the basal planes. The angle β corresponds to the stage tilt between the two STEM images and is indicated in the text. (**C**) Extracted experimental thermal diffusivity (20-μm pitch) D_φ_ as a function of angle φ_M4PP_ between 0 and 90° (for scan site 1). The solid line corresponds to the analytical expression from Eq. 2. (**D**) Extracted thermal diffusivity (20-μm pitch) for scan site 2 as a function of angle φ_M4PP_ between 0° and 90°. The crystallographic direction shown in (B) and (C) corresponds to the scan angles 0°, 50°, and 90°. The complete relationship between thermal diffusivity and crystallographic direction across all scanned angles is detailed in the Supplementary Materials.

Figure 1 (A and B) shows the thermal diffusivity measured with 20-μm pitch, with the collinear line of electrode contacts oriented along the *X* and *Y* axes, respectively, i.e., M4PP||X and M4PP||Y. Figure 1C shows an inverse pole figure (IPF) map of the grain orientation obtained by EBSD, and Fig. 1D shows an optical micrograph of the same region. (See Materials and Methods and the Supplementary Materials for more information on EBSD). We observed a good correlation between spatial features obtained with M4PP, EBSD, and an optical microscope. The results further show a lower thermal diffusivity for M4PP||X in regions where (001), corresponding to the *c* axis, is parallel to X; an important insight that is further quantified in Figs. 2 and 3. Our measurements (Fig. 1) closely align with thermal diffusivity values reported in literature, which are ~1.55 and 0.75 mm^2^/s (*49*–*54*), perpendicular and parallel to the *c* axis, respectively.

Recently, a combination of microscale-level, spatially resolved FDTR and EBSD has been used to explore the local thermal conductivity near GBs in isotropic SnTe (*22*). While this technique enables excellent thermophysical measurements, it is limited by the accuracy of calibration. In contrast, the current method offers a notable advancement in calibration-free measurements with minimal sample preparation.

As indicated by Eq. 1 (see Materials and Methods), the M4PP method is scalable, and the corresponding thermal diffusivity maps obtained using different electrode pitches, such as 10, 20, and 30 μm, yield similar results for the individual grain orientation, as shown in the Supplementary Materials together with the raw data of the measured phase. The amplitude of the thermoelectric signal measured with M4PP increases as electrode pitch decreases. Thus, a smaller electrode pitch improves the signal-to-noise ratio, but simultaneously relative geometrical errors increase, suggesting that the optimal choice of electrode pitch should be further explored to improve precision and resolution (see figs. S8 to S10).

To further quantify the anisotropic thermal diffusivity of Bi_2_Te_3_ and confirm the robustness of our technique, we investigated different regions within different grains as well as the thermal diffusivity in the proximity to and across GBs. Figure 2A shows a polarized optical microscope image of Bi_2_Te_3_ sample with the position of line scans for two probe orientations, i.e., perpendicular (M4PP||X) and parallel (M4PP||Y) to the GBs. Figure 2B shows the corresponding EBSD image of different grain orientations with the corresponding IPF notations. Figure 2C shows a 500-μm line scan (step size, 5 μm) of the local thermal diffusivity measured with the M4PP parallel to the GBs (M4PP||Y). The gray and yellow lines in Fig. 2 (C and D) indicate the reference thermal diffusivity values taken from literature perpendicular and parallel to *c* axis directions (*49*, *51*), respectively. These values originate from a previous study and include a ± 5% error range. The red line represents the bulk thermal diffusivity of the Bi_2_Te_3_ sample *D* = 0.94 mm^2^/s (±3%), which was measured using LFA; see the Supplementary Materials (fig. S7) for more information. The thermal diffusivity measured by M4PP appears fairly uniform across the line scan in Fig. 2C but with microscale variation illustrating the method’s sensitivity to different smaller grains encountered in region X ∈ ([350; 500] μm) as seen in Fig. 2 (A and B).

Figure 2D shows the thermal diffusivity measured with probes oriented perpendicular to GBs (M4PP||X). The values of the thermal diffusivity are observed to switch between literature values for lateral to (gray strip) and parallel to (yellow ribbon) the *c* axis. The spatial position of transitions in the thermal diffusivity correlates well with GB positions captured by EBSD in Fig. 2B. The lateral thermal diffusivity [averaged from Fig. 2C in the region X ∈ ([0; 200] μm)] is *D* = 1.55 mm^2^/s, which is in qualitatively excellent agreement with the lateral thermal diffusivity of 1.55 ± 5% mm^2^/s reported in the literature (*49*–*54*).

We note that the bulk thermal diffusivity value, denoted by the red line obtained through LFA, is lower than the thermal diffusivity value perpendicular to the *c* axis but exceeds the parallel to the *c* axis value for the Bi_2_Te_3_ sample measured using M4PP. The bulk thermal diffusivity measurements using LFA seem to capture the average thermal diffusivity measurement across different grain orientations and are dominated by *c* axis contribution (*27*). As expected, different grain textures in the sample resulted in different thermal diffusivity values, as illustrated in Fig. 2D. Specifically, the thermal diffusivity measured on the left and right side of the middle grain was 1.46 ± 0.05 mm^2^/s. The thermal diffusivity parallel to *c* axis was found to be 0.72 mm^2^/s; see Fig. 2D. The sensitivity of our thermal diffusivity measurements to slight changes in grain orientation is described and identified further in Fig. 3.

Encouraged by the remarkable correlation observed between measured thermal diffusivity and the local crystallographic orientation, we took a more detailed look at the expected local heat transport for different crystallographic orientations. We denote the perpendicular and parallel to *c* axis thermal diffusivities as *D*_⊥_ and *D*_∥_, respectively. We performed an additional experiment to determine the thermal diffusivity along any arbitrary direction as described by Eq. 2 (see Materials Methods). Figure 3A illustrates two locations where measurements were performed (sites 1 and 2).

At each site, 10 measurements were performed for each probe orientation, ranging from 0° to 90° in increments of 10°. The angle between the projection of crystal *c* axis and the line of observation, φ_M4PP_, is shown in Fig. 3A, so that at “site 1,” it almost coincides with φ ≈ φ_M4PP_ − φ_0_ with a small offset φ_0_. Figure 3B shows the EBSD corresponding to the optical scan site depicted, and colors correspond to IPF||Z. The EBSD has been used to provide the exact crystallographic direction for sites 1 and 2. Atomic resolution HAADF-STEM images were acquired on two grains from prepared lamella corresponding to site 1 and 2, respectively, as shown in Fig. 3B. The details of lamella preparation for HAADF-STEM can be found in Materials and Methods and the Supplementary Materials. The strong correlation between EBSD and the crystal orientation determined by STEM provides accurate determination of the thermal diffusivity dependency on the crystallographic orientation in our crystal. The angle β corresponds to the microscope stage tilt angle of 14.24° between the two STEM images.

Figure 3C shows the results of the measurements for site 1. At φ_M4PP_ = 0° the collinear line of the M4PP nearly aligns with the *X* axis (M4PP||X) and at φ_M4PP_ = 90° the M4PP aligns well with Y (M4PP||Y). The measured thermal diffusivity is found to change from the direction parallel to *c* axis (M4PP||Y) (*D* = 0.72 ± 0.03 mm^2^/s) to the direction perpendicular to the *c* axis (M4PP||X) (*D* = 1.6 ± 0.05 mm^2^/s). These two extremes correspond to relative agreement with the few available literature values of principal thermal diffusivities 0.74 and 1.55 mm^2^/s, respectively, reported for Bi_2_Te_3_ (*49*, *51*–*54*). Although we have obtained excellent agreement between our measured values and the literature, one has to appreciate that the absolute values taken from the literature for the Bi_2_Te_3_ may exhibit variations in the compositions depending on their purity levels and synthesis techniques (*54*). Comparing reference values is challenging, particularly since thermal diffusivity/conductivity is sensitive to variations in growth temperature, method, impurities, and microstructure (*54*). Table S4 summarizes thermal conductivity values from three sources, demonstrating their close similarity.

The error bars in Fig. 3C represents the SD from measurements repeated 10 times at each angle. For site 1, the [001] direction of the crystal is almost in-plane, having ϕ = 15° (EBSD) for M4PP||X while ϕ = 89° (EBSD) for M4PP||Y. Similar measurements were conducted on site 2, as shown in Fig. 3D. These measurements indicate a constant thermal diffusivity with an average of *D* = 1.55 ± 0.03 mm^2^/s for all scan angles, corresponding to a perpendicular direction component with respect to the *c* axis of the crystal, i.e., the [001] direction, which is always pointing out of the plane with respect to sample surface for site 2. On the basis of the measurements at site 2 (see Fig. 3D), it is reasonable to assess measurement precision as the mean relative SD of the measured thermal diffusivity. This comprises 10 repeated measurements at all 10 probe orientations, with outliers removed using a 50% median filter. This results in a measurement yield of 99% and a relative SD of 2.5% under near-ideal conditions—specifically, with no visible defects or proximity to GBs.

These results illustrate a simple but powerful way to capture accurately the correlation between grain orientation, which is emphasized by the excellent agreement between the measured thermal diffusivity values and the fitted ones (see Fig. 3C, black line). More detailed information in table S2 shows the complete set of directions [hkl] and their corresponding thermal diffusivity extracted for sites 1 and 2. Currently, except for our method, no other available techniques enable the identification of this particular relationship from a single sample alone without any sample calibration and surface preparation.

We simultaneously measured the electrical resistance through the first harmonic four-point voltage (*40*–*42*, *55*). Similar to the evaluation of anisotropic thermal diffusivities, we determine the perpendicular (σ_⊥_) and the parallel (σ_∥_) electrical conductivities (see fig. S16) in relation to the crystallographic *c* axis, which are presented in table S3 (see the Supplementary Materials), along with thermal diffusivities and the anisotropy ratios *D*_⊥_/*D*_∥_ and σ_⊥_/σ_∥_. These results illustrate the ability of the method to capture locally the orientation-dependent electro-thermal properties in one single measurement.

In addition to Bi_2_Te_3_, we tested our methodology on another classic example of a thermoelectric material, Sb_2_Te_3_ (see fig. S15). The thermal diffusivity measurements obtained through our technique align well with the sparse data available (*54*) in existing literature on this material.

The search for efficient thermoelectric materials continues, and their efficiency depends on the figure of merit *zT* = *T*σα^2^/(κ_*e*_ + κ_*p*_), where α is Seebeck coefficient of the materials, σ is electrical conductivity, and κ_*e*_ and κ_*p*_ are electronic and phononic contributions to total thermal conductivity, respectively (*1*). Thus, knowledge of the relative contributions of the electrons and phonons to the total thermal conductivity of semiconductors or semimetals is not only of fundamental theoretical interest but also of importance in selecting materials. However, detailed measurements of the thermal conductivity at any crystal direction are quite challenging.

**Fig. 4.**
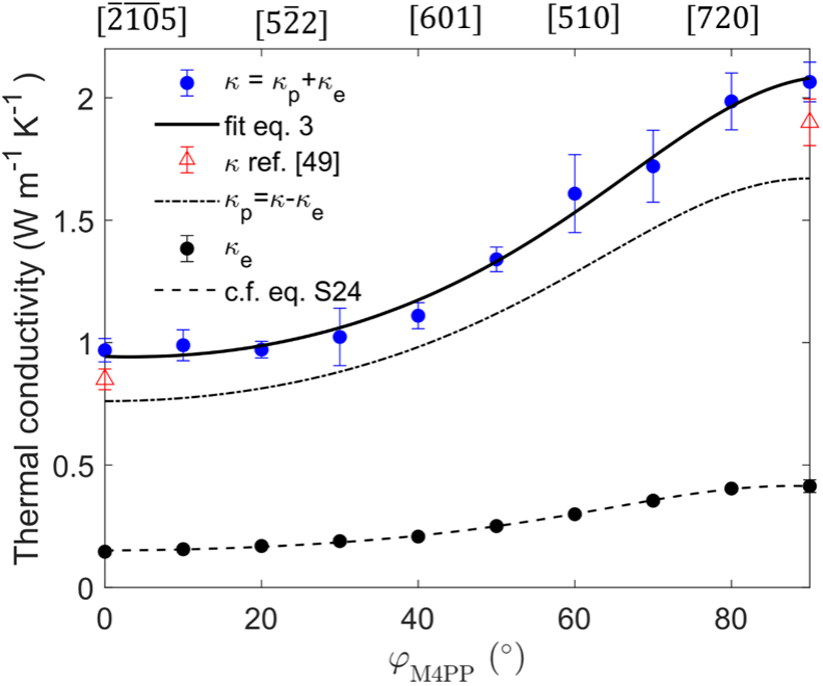
Various thermal conductivity contributions corresponding to different crystallographic orientations. Total thermal conductivity (blue filled circle) of Bi_2_Te_3_ for different crystallographic directions converted from thermal diffusivity measurements for site 1 (Fig. 3). Solid black curves correspond to the fit of the data using Eq. 3. The measured data were used to extract the tensor element of total thermal conductivity of Bi_2_Te_3_. The literature value of the total thermal conductivity for the in- and out- of a plane is shown by open triangles. Using the electrical conductivity (see fig. S16) and the WF relation, the resulting experimental electronic thermal conductivity κ_e_ is shown by black filled symbols (error within symbol) and dashed line for curve c.f. eq. S24. The phononic thermal conductivity, κ_p_, is extracted using the relation κ_p_ = κ − κ_e_ and plotted as dashed-point line for different crystallographic directions. The crystal direction where these measurements were carried out are shown on top *X* axis (the complete table of the crystallographic directions can be found in table S3).

Figure 4 shows the total measured thermal conductivity (κ = κ_*e*_ + κ_*p*_) of Bi_2_Te_3_ at different crystallographic directions for site 1 (see Fig. 3) along with κ_*e*_ and κ_*p*_ at different crystallographic directions, neglecting the contribution of bipolar effects (*27*, *44*). Using the measured electrical conductivity tensor for Bi_2_Te_3_ (see table S3), we estimate the electronic thermal conductivity (κ_e_) using the Wiedemann-Franz (WF) relation (see Materials and Methods). Last, the phononic thermal conductivity (κ_*p*_) is extracted from (κ_*p*_ = κ − κ_*e*_). The out-of-plane phononic thermal conductivity κ_*p*∥_ is found to be lower than the in-plane κ_*p*⊥_ phononic thermal conductivity. This is probably due to the different anharmonicities and atom vibration frequencies along the two directions (*50*, *56*) and the presence of van der Waals bonds parallel to the basal planes, as shown in fig. S4 and Fig. 3B, which affect phonon mean free paths (*57*, *58*). The substantial difference in anharmonicity can be attributed to the distinct bond properties present in the layered structure of Bi_2_Te_3_ (*59*).

## DISCUSSION

Overall, our local measurements of the in- and out-of-plane thermal conductivity agree with predicted and measured macroscopic values reported in the literature (open symbols cf. Fig. 4) (*27*, *44*, *49*–*51*, *54*, *56*, *60*). The intralayer bonds of Bi_2_Te_3_ are predominantly covalent, while the interlayer bonds are a combination of both electrostatic and van der Waals interactions, resulting in hybrid bonds (*50*). These results indicate that the total thermal conductivity (κ) is dominated by lattice contribution for both in- and out-of-plane directions. To summarize, we have used the M4PP as a case study to estimate electronic and phononic thermal conductivity for any crystal direction. Furthermore, the measured ratio of σ/κ multiplied by Lorenz number and temperature at different crystallographic directions is illustrated in fig. S17. It shows a peak around 90°, which may open the door to previously unidentified ways for optimizing and screening high-performing thermoelectric materials in a specific crystallographic direction. Currently, our technique can perform measurements at room temperature (tool limitation) and is limited to electrically conducting samples.

In this study, we have eliminated errors in thermal diffusivity measurements caused by cold finger effects, an issue not addressed in previous work. In addition, our approach operates at a single frequency and single current, reducing measurement time to <1 min per thermal diffusivity measurement and enabling calibration-free, large-area mapping. This paper therefore represents a substantial advancement, opening possibilities for faster and more precise anisotropic thermal diffusivity measurements of thermoelectric materials without the need for calibration or sample preparation (e.g., metal layer deposition).

In conclusion, we show that it is possible to directly image the local thermal diffusivity and the thermal conductivity across different grain orientations as a function of crystallographic direction. This framework provides insights into the possibility of performing detailed material characterization of orientation-dependent thermal diffusivity, eliminating the need for calibration, and accelerating the measurement process by eliminating the deposition of additional gold/metal layer on the sample. These factors are a prerequisite to redefining heat management strategies in electronics and thermoelectric energy harvesting apparatus, capitalizing on the distinctive attributes of GBs and the anisotropic properties of individual grains, paving the way for unprecedented advancements in the field.

## MATERIALS AND METHODS

### Materials fabrication

Bi_2_Te_3_ and Sb_2_Te_3_ ingots were prepared from pure Bi, Sb, and Te granules that were weighted and mixed in the appropriate molar ratios and melted in evacuated and sealed quartz ampoules. The melting process included heating up to 980°C and dwelling for approximately 500 min. Further details are provided in the Supplementary Materials. This process resulted in the growth of larger textured polycrystalline samples, which were subsequently subjected to comprehensive characterization using the below techniques.

### Laser flash measurement

The thermal diffusivity of the prepared pellets was measured at room temperature in an air environment using the LFA technique. This measurement was performed in a MicroFlash LFA-457 system manufactured by Netzsch GmbH, Selb, Germany. The instrumental accuracy of this technique was ±3%. For more details, refer to the Supplementary Materials.

### Crystal structure analysis

The crystal structure analysis was carried out using a Rigaku Miniflex II x-ray diffractometer. This instrument covered an angular range of 20° < 2θ < 80°, with a resolution of 0.01° and a scanning rate of 0.2°/min. For further details, please consult the Supplementary Materials.

### Surface imaging and spectroscopy

Surface imaging, along with elemental and crystallographic analysis, was conducted using a Zeiss Ultra Plus high-resolution scanning electron microscope (SEM). The SEM operated at an acceleration voltage of 4 kV and was equipped with an energy dispersive x-ray spectroscopy (EDS) detector (Oxford SDD EDS detector), operated at 8 kV. EBSD was performed using a Zeiss Sigma 300 SEM having an Oxford c-nano EBSD detector operated at 20 kV. Additional information on sample preparation for EBSD can be found in the Supplementary Materials.

### Electron microscopy characterization

A focused ion beam (FIB) lamella was prepared from the same location as the M4PP measurement (see fig. S19). STEM imaging was acquired in HAADF on a Titan 60-300 aberration corrected microscope operated at 300 kV with a convergence semi-angle of 21 mrad and a collection of 38 to 215 mrad. TEM data were denoised using deep convolution neural networks to restore single-shot electron microscopy images (*61*).

### Thermal diffusivity measurement

We conducted local thermal diffusivity measurements using a CAPRES A301 microRSP-tool that was equipped with an internal lock-in amplifier. To perform these measurements, we used a probe called L10PP, which has 10 L-shaped electrodes in an equidistant design with a pitch of 10 μm (see fig. S2 for optical image of M4PP). In the current M4PP setup, each electrode exerts an extremely small contact force of around 10 ± 5 μN, i.e., about ~10^5^ times smaller force than conventional four-point probes (*62*, *63*). The small contact force allows for nondestructive measurements (*62*) with “zero penetration” (*64*).

This probe was constructed using polysilicon and coated with a 100-nm Ni layer to serve as the current carrier. We used this probe to measure and detect the second harmonic phase delay at a fixed frequency of 385.7 Hz, with a measurement current, optimized at 1.3- and 2-mA root mean square to ensure measurement precision. In M4PP measurements, the measured second harmonic voltage amplitude depends on the ratio of the Seebeck coefficient and thermal conductivity (*43*). However, the measured second harmonic phase only depends on a material’s thermal conductivity.

To obtain thermal diffusivity values, we used a technique involving six four-point measurements, as described by eq. S16. These values were then averaged for the six-mirror configuration using eq. S7. We have used the so called “triplet” scheme that reduces the complexity of measurement results using a set of five electrodes and configuration switching (*65*). A relatively large current is forced between two electrodes, *m* and *n*, giving rise to Joule heating, while a complex thermoelectric voltage at second harmonic, V~2ωpqmn, where *n*, *m*, *p*, *q* ∈ {1, 2, 3, 4, 5}, is measured with a lock-in technique between electrodes *p* and *q* (see more information in the Supplementary Materials). Equation 1 describes the relationship between the measured ratio of triplets and thermal diffusivity, for a sample represented by a half-space with equidistant collinear electrodes placed on the surface.

Γ1=V~2ω2413+V~2ω2415−V~2ω2435V~2ω3412+V~2ω3415−V~2ω3425=3ζ2−132ζ−1,ζ=e(i2ωD)s (1)

Here, *i* is the imaginary unit (*i*^2^ = −1), *s* is the electrode separation (pitch) in micrometers, and ω is the angular frequency of applied current. The procedure of determining the thermal diffusivity does not depend on the nature and shape of the point-like contact. Furthermore, it does not require any external fitting parameter, and the power dissipation as well as cold finger effect from the point-like heat source cancels out. In addition, the measured second harmonic voltage amplitude depends on the anisotropic Seebeck coefficient (if any anisotropy) of the sample. Our method also eliminates the dependency on the Seebeck coefficient and its anisotropy by using the ratio of two single heater signals to measure the second harmonic phase delay and, thereby, determine thermal diffusivity. The M4PP features an array of 10 cantilever electrodes, and by choosing different combinations of electrodes, it is possible to vary the probe pitch to obtain geometrically equivalent results at an equidistant pitch of 10, 20, and 30 μm, respectively (see theoretical background in the Supplementary Materials for the complete detailed information of the method).

### Angle measurement of M4PP

From a simple geometrical consideration, the thermal diffusivity along any direction can be described as

$$D_{\varphi }={\left[\frac{1}{D_{⊥}}+\left(\frac{1}{D_{∥}}-\frac{1}{D_{⊥}}\right){\text{cos}}^{2}\left(\varphi_{M4\text{PP}}-\varphi_{0}\right){\text{cos}}^{2}\left(\phi \right)\right]}^{-1}$$ (2)

Here, φ_M4PP_ is the angle between the projection of crystal *c* axis and the line of observation, i.e., the direction of heat transport in an experiment. While ϕ is the angle between the *c* axis of the crystal and the projected *c* axis on the sample plane, which can be calculated from EBSD data of the corresponding scan site.

At φ_M4PP_ = 0° and ϕ = 0° the line of observation pertains to the parallel to *c* axis component of the thermal diffusivity *D*_∥_. For φ_M4PP_ = 90°, it relates to the perpendicular to *c* axis component of the thermal diffusivity *D*_⊥_. Here, φ_0_ accounts for an in-plane misalignment of M4PP axis to the projected *c* axis. Further background information and a more general description of this is given in the Supplementary Materials (Theoretical background).

### Thermal conductivity 

The thermal conductivity tensor for Bi_2_Te_3_ can be written as $\kappa =\left\{\begin{array}{ccc} \kappa_{\mathrm{xx}} & 0 & 0\\ 0 & \kappa_{\mathrm{yy}} & 0\\ 0 & 0 & \kappa_{\mathrm{zz}} \end{array}\right\}$, where κ_*xx*_ = κ_*yy*_ ≠ κ_*zz*_. Here, κ_*xx*_ = κ_*yy*_ = κ_⊥_ and κ_zz_ = κ_∥_. Since the density and specific heat of Bi_2_Te_3_ are scalars, we can use the relation κ = ρ*c*_*p*_*D* to convert the measured thermal diffusivity from Fig. 3B (site 1) into thermal conductivity (ρ = 7.6 gcm^−3^, *c*_*p*_ = 165 J kg^−1^ K^−1^) (*66*). We fitted the thermal conductivity (κ_φ_) using Eq. 3 to extract the tensor component κ_⊥_ (2.08 Wm^−1^ K^−1^) and κ_∥_ (0.9 Wm^−1^ K^−1^). The electronic thermal conductivity (κ_*e*_) tensor elements (κ_*e*⊥_ and κ_*e*∥_) were estimated using the extracted electrical conductivity (σ) tensor from the fitted curve (see fig. S16) using WF rule ($\kappa_{e}=\sigma TL$) (*67*), where $L=\frac{\pi^{2}}{3}{\left(\frac{k_{B}}{e}\right)}^{2}$, *k*_*B*_ is Boltzmann’s constant, *e* the unit charge, and *T* the absolute temperature. The thermal conductivity along any direction can be described as

$$\kappa_{\varphi }={\left[\frac{1}{\kappa_{⊥}}+\left(\frac{1}{\kappa_{∥}}-\frac{1}{\kappa_{⊥}}\right)\;{\text{cos}}^{2}\left(\varphi_{M4\text{PP}}-\varphi_{0}\right)\;{\text{cos}}^{2}\left(\phi \right)\right]}^{-1}$$ (3)

The tensor elements of electronic thermal conductivity were determined to be $\kappa_{e⊥}=\sigma_{⊥}TL$ and $\kappa_{e∥}=\sigma_{∥}TL$ at T = 300 K, while the lattice thermal conductivity elements κ_*p*⊥_ and κ_*p*∥_ were estimated from κ_*p*⊥_ = κ_⊥_ − κ_*e*⊥_ and κ_*p*∥_ = κ_∥_ − κ_*e*∥_. We can apply this simple relation since the bipolar contribution to the total thermal conductivity is negligible at room temperature (*27*).

## Supplementary Materials

### This PDF file includes:

Supplementary Text Figs. S1 to S19 Tables S1 to S5 References
