# Supplementary material for: Thermal diffusivity microscope: Zooming in on anisotropic heat transport
Source: Sci Adv. 2025 Feb 26;11(9):eads6538. doi: 10.1126/sciadv.ads6538 (PMC11864195; doi:10.1126/sciadv.ads6538)
Supplement: Supplementary file 1 — Supplementary Text Figs. S1 to S19 Tables S1 to S5 References [file sciadv.ads6538_sm.pdf]

Science Advances
AAAS

Supplementary Materials for

Thermal diffusivity microscope: Zooming in on anisotropic heat transport

Neetu Lamba *et al.*

Corresponding author: Neetu Lamba, neetura@dtu.dk; Nini Pryds, nipr@dtu.dk

Sci. Adv. **11**, eads6538 (2025)
DOI: 10.1126/sciadv.ads6538

This PDF file includes:

Supplementary Text
Figs. S1 to S19
Tables S1 to S5
References

1 Theoretical Background

1.1 Thermal field from a point heat source

The temperature field $\tilde{T}$, due to a harmonic excitation at angular frequency 2ω, in a material, is governed by the heat continuity equation $\mathrm{i}2\omega\varrho c\tilde{T} = \nabla\cdot\bar{\kappa}\nabla\tilde{T}$, where $\mathrm{i}=\sqrt{-1}$ is the imaginary unit, $\bar{\kappa}$ is the thermal conductivity tensor, ϱ is the mass density, c the specific heat capacity at constant pressure such that ϱc is the heat capacity per volume, and $\tilde{T}$ is the complex temperature, while the instantaneous physical temperature is $T = \mathrm{Re}\tilde{T}$. According to Onsager's relations, the thermal conductivity tensor is symmetric, and if the chosen coordinates (x,y,z) are parallel to the principal axis of thermal conductivity, the thermal conductivity tensor becomes diagonal with entries κ_x, κ_y and κ_z (principal thermal conductivities). A simple coordinate transformation to $u_x = x\sqrt{\kappa_0/\kappa_x}$, $u_y = y\sqrt{\kappa_0/\kappa_y}$, and $u_z = z\sqrt{\kappa_0/\kappa_z}$ transforms the right-hand side of the heat continuity equation to a simple Laplacian in the transformed coordinates, i.e., the heat continuity equation becomes $\mathrm{i}2\omega\varrho c\tilde{T} = \kappa_0\nabla_u^2\tilde{T}$, which simplifies solution of the heat equation.

The spherically symmetric solution in transformed coordinates is

$$\tilde{T}_{2\omega} = \frac{\mathcal{B}}{u}\exp\left(-\sqrt{\frac{\mathrm{i}2\omega}{\kappa_0}}u\right), \tag{S1}$$

where $u=\sqrt{u_x^2+u_y^2+u_z^2}=\sqrt{x^2\kappa_0/\kappa_x+y^2\kappa_0/\kappa_y+z^2\kappa_0/\kappa_z}$, and κ_0 is a completely arbitrary scaling factor with the dimension of a thermal conductivity(*68*). For convenience we take $\kappa_0 = \sqrt[3]{\kappa_x\kappa_y\kappa_z}$ which is the geometric mean of the principal thermal conductivities. We note that $\sqrt{\mathrm{i}} = (1+\mathrm{i})/\sqrt{2}$ such that the argument of the exponential has equal real and imaginary magnitudes, we shall, however, for compactness of notation not expand $\sqrt{\mathrm{i}}$ in the following.

The constant of integration $\mathcal{B}$ is determined by the conditions at origo $u=0$. If the harmonic heating power $\tilde{P}_{2\omega}$ is injected at a point $(u=0)$ on the sample surface $\mathcal{B}=\frac{\tilde{P}_{2\omega}}{2\pi}\sqrt{\frac{\kappa_0}{\kappa_x\kappa_y\kappa_z}}=\frac{\tilde{P}_{2\omega}}{2\pi\kappa_0}$, while injection of the power in the surface of a small hemisphere (of radius b, in u-coordinates) at $u=0$ results in $\mathcal{B}=\frac{\tilde{P}_{2\omega}}{2\pi\kappa_0}\frac{\exp\left(\sqrt{\frac{\mathrm{i}2\omega\varrho c}{\kappa_0}}b\right)}{1+\sqrt{\frac{\mathrm{i}2\omega\varrho c}{\kappa_0}}b+\frac{Y_p}{2\pi\kappa_0 b}}$, where Y_p is the thermal admittance of the heating probe-pin as seen from the sample surface, which is used here to account for a so-called "cold-finger" effect if necessary. From the expressions for $\mathcal{B}$ we see that its phase $\arg\mathcal{B}$ includes not only the phase of the injected power $\arg\tilde{P}_{2\omega}$, but also contributions from the finite size of the injection point and the "cold-finger".

1.2 Temperature along an arbitrary line

Our main interest is the temperature along a line on the surface of a sample. A completely arbitrary line can be described by parameters (λ,θ,φ) where λ is the position

along the line while the direction of the line is described by the angles (θ,φ), such that $x = \lambda\cos\theta\sin\varphi$, $y = \lambda\sin\theta\sin\varphi$, $z = \lambda\cos\varphi$. Therefore, we have $u^2 = u_x^2 + u_y^2 + u_z^2 = \kappa_0\lambda^2\left(\frac{\cos^2\theta\sin^2\varphi}{\kappa_x} + \frac{\sin^2\theta\sin^2\varphi}{\kappa_y} + \frac{\cos^2\varphi}{\kappa_z}\right)$, and the corresponding temperature along this arbitrary line from a point source (heating power $\tilde{P}_{2\omega}^{(0)}$) at λ_0 on the line becomes

$$\tilde{T}_{2\omega}(\lambda_0,\lambda) = \mathcal{A}_{\theta,\varphi}^{(0)}\frac{\exp\left(-\sqrt{\frac{\mathrm{i}2\omega}{D_{\theta,\varphi}}}|\lambda-\lambda_0|\right)}{|\lambda-\lambda_0|}, \tag{S2}$$

where the pre-factor $\mathcal{A}_{\theta,\varphi}^{(0)}$ is

$$\mathcal{A}_{\theta,\varphi}^{(0)} = \frac{\tilde{P}_{2\omega}^{(0)}}{2\pi\sqrt{\kappa_x\kappa_y\kappa_z\left[\left(\frac{\cos^2\theta}{\kappa_x} + \frac{\sin^2\theta}{\kappa_y}\right)\sin^2\varphi + \frac{\cos^2\varphi}{\kappa_z}\right]}}. \tag{S3}$$

We have also used the thermal diffusivity $D_{\theta,\varphi}$ in the direction (θ,φ), i.e.,

$$D_{\theta,\varphi} = \left[\left(\frac{\cos^2\theta}{D_x} + \frac{\sin^2\theta}{D_y}\right)\sin^2\varphi + \frac{\cos^2\varphi}{D_z}\right]^{-1}, \tag{S4}$$

where $D_x = \kappa_x/(\varrho c)$, $D_y = \kappa_y/(\varrho c)$, and $D_z = \kappa_z/(\varrho c)$ are the thermal diffusivities along the principal directions. A simple inspection shows that $\mathcal{A}_{\theta,0}^{(0)} = \tilde{P}_{2\omega}^{(0)}/\left(2\pi\sqrt{\kappa_x\kappa_y}\right)$ and $D_{\theta,0} = D_z$, and $\mathcal{A}_{0,\pi/2}^{(0)} = \tilde{P}_{2\omega}^{(0)}/\left(2\pi\sqrt{\kappa_y\kappa_z}\right)$ and $D_{0,\pi/2} = D_x$ etc. as expected.

An important class of materials have principal conductivities $\kappa_x = \kappa_y = \kappa_\perp$ and $\kappa_z = \kappa_\|$ and thus principal diffusivities $D_x = D_y = D_\perp$ and $D_z = D_\|$, where $\perp$ indicates perpendicular to and $\|$ parallel to the c-axis. For these materials the expressions, $\mathcal{A}_{\theta,\varphi}^{(0)}$ and $D_{\theta,\varphi}$, becomes independent on θ and simplify to

$$\mathcal{A}_{\theta,\varphi}^{(0)} = \frac{\tilde{P}_{2\omega}^{(0)}}{2\pi\sqrt{\kappa_\perp^2\kappa_\|\left(\frac{\sin^2\varphi}{\kappa_\perp} + \frac{\cos^2\varphi}{\kappa_\|}\right)}}. \tag{S5}$$

and

$$D_{0,\varphi} = \left(\frac{\sin^2\varphi}{D_\perp} + \frac{\cos^2\varphi}{D_\|}\right)^{-1} = \left(\frac{1-\cos^2\varphi}{D_\perp} + \frac{\cos^2\varphi}{D_\|}\right)^{-1}, \tag{S6}$$

where we emphasize that the angle φ is the angle between the line of interest (i.e., the line of the probe) and the z-axis (i.e., the c-axis) of the crystal.

1.3 Temperature differences

The harmonic temperature difference $\Delta\tilde{T}_{2\omega}(\lambda_0,\lambda_p,\lambda_q)$ from a single heater (at λ_0) between two points, λ_p and λ_q on the λ-axis is

$$\Delta\tilde{T}_{2\omega}(\lambda_0,\lambda_p,\lambda_q) = \tilde{T}_{2\omega}(\lambda_0,\lambda_p) - \tilde{T}_{2\omega}(\lambda_0,\lambda_q), \tag{S7}$$

and in principle, a very good proxy for this temperature difference can be measured, by use of the Seebeck effect, if nominally identical electrodes are placed at the points λ_p and λ_q, which results in the harmonic Seebeck voltage difference $\tilde{V}_{2\omega}(\lambda_0,\lambda_p,\lambda_q)$

$$\tilde{V}_{2\omega}(\lambda_0,\lambda_p,\lambda_q) = -(\alpha_s - \alpha_e)\,\Delta\tilde{T}_{2\omega}(\lambda_0,\lambda_p,\lambda_q), \tag{S8}$$

where α_s and α_e are the Seebeck coefficients of the sample and the electrodes, respectively.

1.4 Short notation

From now onwards, we will use the short notation for describing the exact combination of experimental configurations, it is convenient to reduce the notation into a compact format, here we define ${}^{0}_{pq}\Delta\tilde{T}_{2\omega} = \Delta\tilde{T}_{2\omega}(\lambda_0,\lambda_p,\lambda_q)$, ${}^{0}_{pq}\tilde{V}_{2\omega} = \tilde{V}_{2\omega}(\lambda_0,\lambda_p,\lambda_q)$, ${}^{0}_{p}\tilde{T}_{2\omega} = \tilde{T}_{2\omega}(\lambda_0,\lambda_p)$ and ${}^{0}_{q}\tilde{T}_{2\omega} = \tilde{T}_{2\omega}(\lambda_0,\lambda_q)$

1.5 Collinear equidistant probes

Assuming that the harmonic Seebeck voltages are measured with a collinear equidistant probe with N electrodes, i.e., a probe with $\lambda_{j+1} - \lambda_j = s$, where s is the probe pitch and $j \in [1,2...N]$. With this probe, we can make two measurements ${}^{1}_{24}\tilde{V}_{2\omega}$ and ${}^{1}_{34}\tilde{V}_{2\omega}$; then taking the ratio of both

$$\Gamma_1 = \frac{{}^{1}_{24}\tilde{V}_{2\omega}}{{}^{1}_{34}\tilde{V}_{2\omega}} = \frac{{}^{1}_{2}\tilde{T}_{2\omega} - {}^{1}_{4}\tilde{T}_{2\omega}}{{}^{1}_{3}\tilde{T}_{2\omega} - {}^{1}_{4}\tilde{T}_{2\omega}} = \frac{\frac{{}^{1}_{2}\tilde{T}_{2\omega}}{{}^{1}_{4}\tilde{T}_{2\omega}} - 1}{\frac{{}^{1}_{3}\tilde{T}_{2\omega}}{{}^{1}_{4}\tilde{T}_{2\omega}} - 1} \tag{S9}$$

$$= \frac{\frac{|\lambda_4-\lambda_1|}{|\lambda_2-\lambda_1|}\zeta^2 - 1}{\frac{|\lambda_4-\lambda_1|}{|\lambda_3-\lambda_1|}\zeta - 1} = \frac{\frac{3}{1}\zeta^2 - 1}{\frac{3}{2}\zeta - 1}, \text{ with } \zeta = \exp\left(\sqrt{\frac{\mathrm{i}2\omega}{D_{\theta,\varphi}}}s\right), \tag{S10}$$

eliminates the dependency on Seebeck coefficients as well as the detailed conditions at the heat injection point, i.e., the comprehensive expression for $\mathcal{B}$ and $\mathcal{A}^{(1)}_{\theta,\varphi}$. By solving the experimental phase of ratios Γ_1 with diffusivity $D_{\theta,\varphi}$ as a free parameter, we can extract the thermal diffusivity of the sample along the λ-axis free from any heat losses and any external inputs. The equation can also be inverted analytically, i.e.,

$$D_{\theta,\varphi} = \frac{\mathrm{i}2\omega s^2}{\ln^2\left(\frac{1}{4}\left(\Gamma_1 + \sqrt{\Gamma_1^2 - 16\frac{\Gamma_1 - 1}{3}}\right)\right)}. \tag{S11}$$

A similar set of measurements ${}^{4}_{31}\tilde{V}_{2\omega}$ and ${}^{4}_{21}\tilde{V}_{2\omega}$, with λ_4 as the heat source position,

leads to the ratio

$$\Gamma_4 = \frac{{}^{4}_{31}\tilde{V}_{2\omega}}{{}^{4}_{21}\tilde{V}_{2\omega}} = \frac{{}^{4}_{3}\tilde{T}_{2\omega} - {}^{4}_{1}\tilde{T}_{2\omega}}{{}^{4}_{2}\tilde{T}_{2\omega} - {}^{4}_{1}\tilde{T}_{2\omega}} = \frac{\frac{{}^{4}_{3}\tilde{T}_{2\omega}}{{}^{4}_{1}\tilde{T}_{2\omega}} - 1}{\frac{{}^{4}_{2}\tilde{T}_{2\omega}}{{}^{4}_{1}\tilde{T}_{2\omega}} - 1} = \tag{S12}$$

$$= \frac{\frac{3}{1}\zeta^2 - 1}{\frac{3}{2}\zeta - 1}, \text{ with } \zeta = \exp\left(\sqrt{\frac{\mathrm{i}2\omega}{D_{\theta,\varphi}}}s\right), \tag{S13}$$

which eliminates the dependence on $\mathcal{A}^{(4)}_{\theta,\varphi}$, and from this ratio the diffusivity

$$D_{\theta,\varphi} = \frac{\mathrm{i}2\omega s^2}{\ln^2\left(\frac{1}{4}\left(\Gamma_4 + \sqrt{\Gamma_4^2 - 16\frac{\Gamma_4 - 1}{3}}\right)\right)}$$

results. Several such independent experimental diffusivities may be used to validate the measurement and the average of these has improved precision compared to the individual measurement.

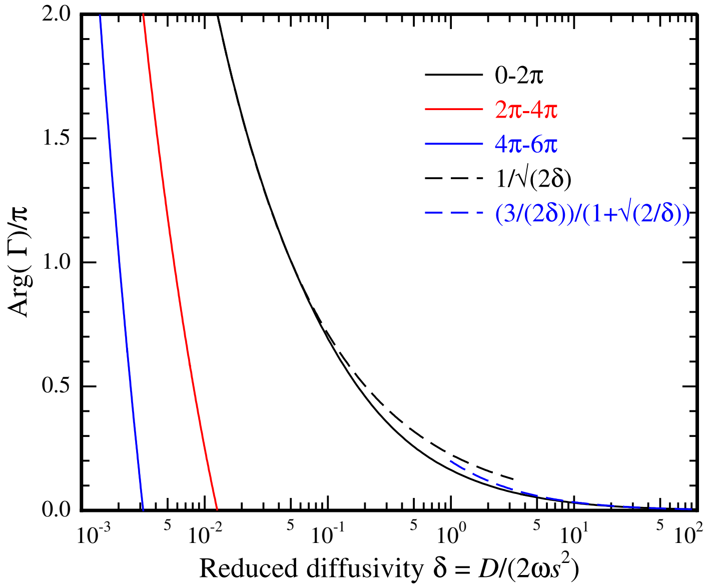

Fig. S1: Phase behavior of Γ. $\arg\Gamma$ plotted as a function of the reduced diffusivity $\delta = D/(2\omega s^2)$ with definition domain of $[0;2\pi]$ for the arg function. The two asymptotic approximations $\arg\Gamma \simeq 1/\sqrt{2\delta}$ and $\arg\Gamma \simeq \frac{3}{2\delta}\Big/\left(1+\sqrt{\frac{2}{\delta}}\right)$ are also shown and agree well with the exact expression.

For a study of the behavior of the phase of the voltage ratio, i.e., $\arg\Gamma$, it is useful to define the reduced diffusivity $\delta = D/\left(2\omega s^2\right)$ and then it follows that

$$\Gamma = \left[\frac{3}{1}\exp\left(2\sqrt{\frac{\mathrm{i}}{\delta}}\right) - 1\right] \Big/ \left[\frac{3}{2}\exp\left(\sqrt{\frac{\mathrm{i}}{\delta}}\right) - 1\right].$$

Now, if $\delta \ll 1$ we have $\Gamma \simeq 2\exp\sqrt{\frac{\mathrm{i}}{\delta}} = 2\exp\left(\frac{1+\mathrm{i}}{\sqrt{2\delta}}\right)$ and it follows that $\arg\Gamma \simeq 1/\sqrt{2\delta}$, while at $\delta \gg 1$ we have $\arg\Gamma \simeq \frac{3}{2\delta}\Big/\left(1+\sqrt{\frac{2}{\delta}}\right)$.

If we restrict $\arg\Gamma$ to the interval $[0;2\pi]$ the function $\arg\Gamma$ has several branches, and the fundamental branch point occurs at $2\pi = \arg\Gamma \simeq 1/\sqrt{2\delta_{\mathrm{min}}}$ and thus $\delta_{\mathrm{min}} \simeq 1/(2\times(2\pi)^2) \simeq 1.2665\times10^{-2}$ is the smallest value of the reduced diffusivity which remains on the fundamental branch of $\arg\Gamma$ as illustrated in Fig. S1. This lower limit on the reduced diffusivity corresponds to the minimum diffusivity $D_{\mathrm{min}} = \omega s^2/(2\pi)^2 = f s^2/(2\pi)$ where $f = \omega/(2\pi)$ is the frequency.

1.6 Practical implementation of point heat sources

In a conventional micro four-point probe (M4PP) measurement using the collinear equidistant probe with $N = 10$ electrodes as described above, an equidistant sub-probe, e.g., electrodes #1–#4, is chosen and a harmonic current $\tilde{I}$ at angular frequency ω is forced through one electrode (e.g., electrode #1) and extracted through another electrode (e.g. electrode #4) while the harmonic difference voltage between two electrodes (e.g., electrodes #2 and #3) is measured, i.e., at the fundamental frequency we have ${}^{14}_{23}\tilde{V}^{(4p)}_{1\omega}$ and this voltage and the four-point transfer-resistance $R^{(4p)}_{1\omega} = {}^{14}_{23}\tilde{V}^{(4p)}_{1\omega}/\tilde{I}$ is directly related to the resistivity and the geometry of sample/probe-pin configuration and is widely used to characterize materials and thin films electrically(*40-42,69*). Note, that no phase shift is expected between current and first harmonic voltage unless the frequency is very high, thus $R^{(4p)}_{1\omega}$ is really a transfer-resistance. The physical current used is $I(t) = I_0\sin(\omega t)$, thus $\tilde{I} = I_0\exp(\mathrm{i}\omega t - \pi/2)$. The injected current $I(t)$ causes a current density $J(\mathbf{r},t) = \frac{I(t)}{2\pi|\mathbf{r}|^2}$ where $\mathbf{r}$ is the position vector from the injection point. The current density causes a joule heating density of $\rho J^2(\mathbf{r},t)$ which, combined with the contact heating, results in a thermal field with a $|\mathbf{r}|^{-1}$ term and a $|\mathbf{r}|^{-2}$ term. The $|\mathbf{r}|^{-2}$ term is directly related to the joule heating density $\rho J^2(\mathbf{r},t)$ and decays rapidly with distance (on a contact radius scale) compared to the $|\mathbf{r}|^{-1}$ term, which is caused by both (sample and contact) heat contributions. It follows that the thermal far field (i.e., several contact radii away) is very well approximated by assuming that all heat is generated at the contact (i.e., ignoring the $|\mathbf{r}|^{-2}$ term in the thermal field).

The current forced through two of the probe pins leads to very localized Joule heating due to contact and sample resistance, such that an M4PP measurement to an excellent approximation realizes two point-heat sources at the sample surface. The instantaneous power deposited at the two points is $P_j(t) = R_{Cj}I^2(t) = R_{Cj}I_0^2\sin^2(\omega t) = \frac{1}{2}R_{Cj}I_0^2(1-\cos(2\omega t))$, $j \in [1,4]$, where the effective contact resistance (R_{C1} and R_{C4}) of probes #1 and #4 may differ. It follows that the heating power has two frequency components, one at DC and one at the second harmonic 2ω which is of interest here, and we may write $\tilde{P}^{(j)}_{2\omega} = \frac{1}{2}R_{Cj}I_0^2\exp(\mathrm{i}2\omega t - \pi)$ for each of the heating probes.

The two heating powers give rise to a four-point probe Seebeck voltage difference

${}^{14}_{23}\tilde{V}^{(4p)}_{2\omega}$ at the second harmonic frequency between the two potential pins; we have

$$ {}^{14}_{23}\tilde{V}^{(4p)}_{2\omega} = {}^{1}_{23}\tilde{V}^{(4p)}_{2\omega} + {}^{4}_{23}\tilde{V}^{(4p)}_{2\omega} \tag{S14} $$

due to the linearity of the heat continuity equation such that superposition is valid. We note that ${}^{14}_{23}\tilde{V}^{(4p)}_{2\omega} = {}^{41}_{23}\tilde{V}^{(4p)}_{2\omega}$.

In our previous study(*65*), we showed that it is possible to reduce the complexity of measurements from two heat sources to one heat source by adding a fifth electrode. Using five electrodes, the second harmonic four-point voltage is measured for three sets of four electrodes (sub-probes). Through a linear combination, the problem reduces to the voltage drop from a single heater i.e., we measure ${}^{14}_{23}\tilde{V}^{(4p)}_{2\omega}$, ${}^{15}_{23}\tilde{V}^{(4p)}_{2\omega}$, and ${}^{45}_{23}\tilde{V}^{(4p)}_{2\omega}$ and using Eq.9 from the ref.(*65*)

$$ {}^{1}_{23}\tilde{V}_{2\omega} = \frac{{}^{14}_{23}\tilde{V}^{(4p)}_{2\omega} + {}^{15}_{23}\tilde{V}^{(4p)}_{2\omega} - {}^{45}_{23}\tilde{V}^{(4p)}_{2\omega}}{2}, \tag{S15} $$

which shows that Eq. S15 leaves us with a single heater at λ_1 and measured Seebeck voltages between electrodes at λ_2 and λ_3 i.e. Eq. S8.

In our current M4PP thermal diffusivity measurement, we have six sets of four-point configurations for extracting a single thermal diffusivity, i.e., corresponding to probe pin ID (1, 3, 4, 5, 6, 9)

$$ {}^{3}_{46}\tilde{V}_{2\omega} = \frac{{}^{35}_{46}\tilde{V}^{(4p)}_{2\omega} + {}^{13}_{46}\tilde{V}^{(4p)}_{2\omega} - {}^{15}_{46}\tilde{V}^{(4p)}_{2\omega}}{2} \text{ and } {}^{3}_{56}\tilde{V}_{2\omega} = \frac{{}^{34}_{56}\tilde{V}^{(4p)}_{2\omega} + {}^{39}_{56}\tilde{V}^{(4p)}_{2\omega} - {}^{49}_{56}\tilde{V}^{(4p)}_{2\omega}}{2}. \tag{S16} $$

By taking the ratio of two ${}^{3}_{46}\tilde{V}_{2\omega}$ and ${}^{3}_{56}\tilde{V}_{2\omega}$ leads to the Eq. S10. To further extract thermal diffusivity from the ratio, the $\arg({}^{3}_{46}\tilde{V}_{2\omega}/{}^{3}_{56}\tilde{V}_{2\omega}) = \arg(\Gamma_1)$ is solved implicitly using Matlab. Similarly, to have better precision, we can average thermal diffusivity from mirror configurations; therefore, below is another set of six configurations corresponding to probe pin ID (1, 3, 4, 5, 6, 9),

$$ {}^{6}_{53}\tilde{V}_{2\omega} = \frac{{}^{46}_{53}\tilde{V}^{(4p)}_{2\omega} + {}^{16}_{53}\tilde{V}^{(4p)}_{2\omega} - {}^{14}_{53}\tilde{V}^{(4p)}_{2\omega}}{2} \text{ and } {}^{6}_{43}\tilde{V}_{2\omega} = \frac{{}^{56}_{43}\tilde{V}^{(4p)}_{2\omega} + {}^{69}_{43}\tilde{V}^{(4p)}_{2\omega} - {}^{59}_{43}\tilde{V}^{(4p)}_{2\omega}}{2}. \tag{S17} $$

The ratio of two ${}^{6}_{53}\tilde{V}_{2\omega}$ and ${}^{6}_{43}\tilde{V}_{2\omega}$ leads to the Eq. S13 and $\arg(\Gamma_4)$ is further used to solve for thermal diffusivity using Matlab. Here, to solve for thermal diffusivity, we have preferred implicit over explicit solutions; the former we tentatively found to be less sensitive to position errors than the explicit solution. While solving implicitly, we make sure to stay on the fundamental solution branch of $\arg\Gamma$ by restricting the diffusivity to values above $D_{\min} = f s^2/(2\pi)$ as defined in Section 1.5. E. g., with a measurement frequency of $f = 385.7\,\mathrm{Hz}$ and a pitch of $s = 10\,\mu\mathrm{m}$ the minimum diffusivity of $D_{\min} \simeq 0.0061\ \mathrm{mm}^2/\mathrm{s}$ results.

An illustration of six four-point measurements is shown below in Fig. S2. Fig. S3 shows a top view image of the measurement taken from the M4PP tool camera, the image shows the probe with 10 L-shaped electrodes of 10 μm pitch engaged with the sample.

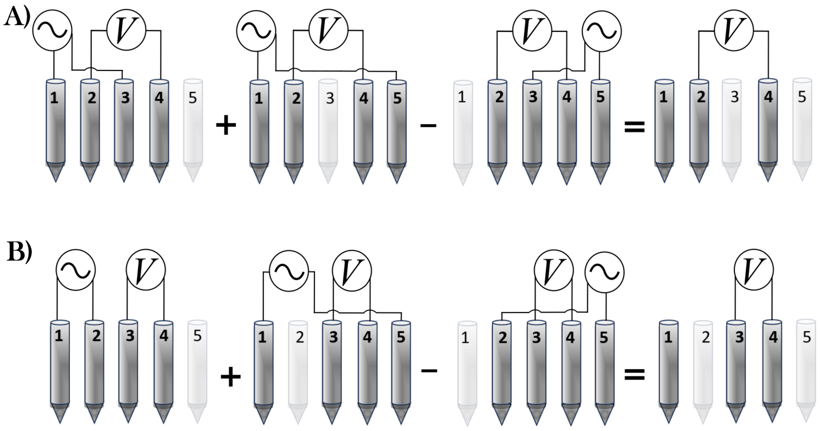

Fig. S2: Schematic of six triplet configuration. Illustration of a set of six (three for each triplet A) and B)) four-point configurations used for extracting one thermal diffusivity value.

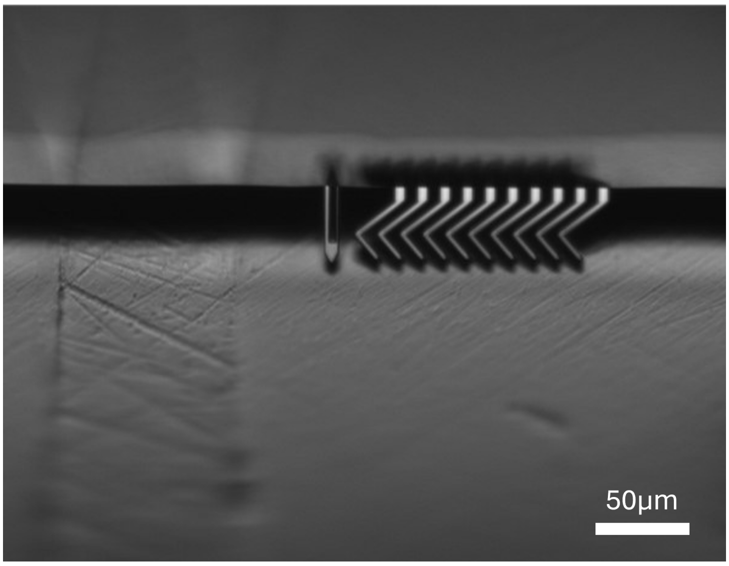

Fig. S3: Overview image of an M4PP measurement. Top view from the M4PP tool camera showing measurement conditions, with the leftmost side indicating the strain gauge for surface detection followed by ten equidistant L-shaped cantilevers for measurement.

1.7 Electrical M4PP resistivity measurements

An analysis similar to that of the thermal analysis, albeit much simpler, leads to expressions for the electrical resistivity ρ_{eff} or conductivity σ_{eff} measured at the first harmonic

frequency. The measurement can be performed simultaneously with the M4PP thermal diffusivity measurement at the second harmonic frequency. Here, for simplicity, the crystal is again assumed to have enough symmetry that the resistivity tensor is symmetric with diagonal elements $\rho_x = \rho_y = \rho_\perp$ and $\rho_z = \rho_{||}$, or an equivalent conductivity tensor with $\sigma_x = \sigma_y = \sigma_\perp = 1/\rho_\perp$ and $\sigma_z = \sigma_{||} = 1/\rho_{||}$, were again subscript $\perp$ indicates the components perpendicular to the c-axis and $||$ the component parallel to the c-axis.

The electrical potential Φ is governed by the current continuity equation $\nabla \cdot \bar{\sigma} \nabla \Phi = 0$, where $\bar{\sigma}$ is the conductivity tensor. By a similar coordinate transformation as that used for the thermal problem, the current continuity equation is converted to a Laplacian, which facilitates solution. The potential along an arbitrary line (λ,θ,φ) from a point current source I at λ_0 is then obtained as

$$\Phi(\lambda_0,\lambda) = \frac{I}{2\pi\,|\lambda-\lambda_0|}\frac{1}{\sqrt{\sigma_\perp\sigma_{||}\sin^2\varphi+\sigma_\perp^2\cos^2\varphi}} = \frac{I}{2\pi\,|\lambda-\lambda_0|\,\sigma_{\text{eff}}}.$$

Thus, the effective conductivity or resistivity are obtained from

$$\sigma_{\text{eff}} = \sqrt{\sigma_\perp\sigma_{||}\sin^2\varphi+\sigma_\perp^2\cos^2\varphi} \text{ and } \rho_{\text{eff}} = \left(\sqrt{\frac{\sin^2\varphi}{\rho_\perp\rho_{||}}+\frac{\cos^2\varphi}{\rho_\perp^2}}\right)^{-1}. \tag{S18}$$

The transfer resistance was measured with an equidistant (pitch s) four-point probe is then $R_A = {}_{23}^{14}\tilde{V}_{1\omega}^{(4p)}/\tilde{I} = \rho_{\text{eff}}/(2\pi s)$ and $R_B = {}_{24}^{13}\tilde{V}_{1\omega}^{(4p)}/\tilde{I} = \rho_{\text{eff}}/(3\pi s)$ in the A- and B-configurations, respectively. By measuring both configurations, the extracted resistivity can be corrected for small position errors, i.e., the probe pins do not contact the surface at the exact intended positions. Position correction to the second order can be obtained with the following equation for bulk measurements,

$$\frac{4}{3}\left(\frac{2\pi sR_A}{\rho_{\text{eff}}}+\frac{2}{7}\frac{\rho_{\text{eff}}}{2\pi sR_A}\right)-\frac{5}{9}\left(\frac{3\pi sR_B}{\rho_{\text{eff}}}+\frac{2}{7}\frac{\rho_{\text{eff}}}{3\pi sR_B}\right) = 1, \tag{S19}$$

where s is the nominal pitch. This procedure improves the validity of the extracted resistivity in particularly at small pitch values. Eq. S19 can be derived as follows: fixing a proper linear combination of (sR_A/ρ_{eff}) and (sR_B/ρ_{eff}) to 1 provides first-order position correction and adding a proper linear combination of $(sR_A/\rho_{\text{eff}})^{-1}$ a nd $(sR_B/\rho_{\text{eff}})^{-1}$ provides position error correction to second order. Eq. S19 is an unpublished result derived in our group in 2010, and its validity has been verified using synthetic data with position noise.

1.8 Practical relations to relevant measurement angles

Let's assume that we have the (unit length) vectors $\hat{\mathbf{X}}$, $\hat{\mathbf{Y}}$, $\hat{\mathbf{Z}}$, $\hat{\mathbf{C}}$, where $\hat{\mathbf{X}}$ and $\hat{\mathbf{Y}}$ form an orthogonal set of vectors in the sample plane, $\hat{\mathbf{Z}}$ is normal to the sample plane, while $\hat{\mathbf{C}}$ is parallel to the c-axis of the crystal. We are interested in the angle φ between the line of the M4PP (vector $\hat{\mathbf{V}}_{\text{M4PP}}$) and the c-axis.

In general we can express any vector in terms of $\hat{\mathbf{X}}$, $\hat{\mathbf{Y}}$, and $\hat{\mathbf{Z}}$, such that $\hat{\mathbf{C}} = (\hat{\mathbf{X}}\cos\varphi_0 + \hat{\mathbf{Y}}\sin\varphi_0)\cos\phi + \hat{\mathbf{Z}}\sin\phi$, where $(\hat{\mathbf{X}}\cos\theta_0 + \hat{\mathbf{Y}}\sin\theta_0)\cos\phi$ is the projection of $\hat{\mathbf{C}}$ on the X-Y-plane, and ϕ is the angle between $\hat{\mathbf{C}}$ and its projection on the X-Y plane. The line of the M4PP is parallel to the vector $\hat{\mathbf{V}}_{\text{M4PP}} = \hat{\mathbf{X}}\cos\varphi_{_{\text{M4PP}}} + \hat{\mathbf{Y}}\sin\varphi_{_{\text{M4PP}}}$ where $\varphi_{_{\text{M4PP}}}$ is the angle between the X-axis and the line of the M4PP. By definition, the angle φ is the angle between $\hat{\mathbf{C}}$ and $\hat{\mathbf{V}}_{\text{M4PP}}$ and thus

$$\begin{aligned}\cos\varphi = \hat{\mathbf{V}}_{\text{M4PP}} \cdot \hat{\mathbf{C}} &= (\cos\varphi_0\cos\varphi_{_{\text{M4PP}}} + \sin\varphi_0\sin\varphi_{_{\text{M4PP}}})\cos\phi \\ &= \cos\phi\cos(\varphi_{_{\text{M4PP}}} - \varphi_0) \end{aligned} \tag{S20}$$

Thus, it is convenient to rewrite the expression for the thermal diffusivity as follows

$$\begin{aligned} D_\varphi &= \left(\frac{1}{D_\perp} + \left(\frac{1}{D_\parallel} - \frac{1}{D_\perp}\right)\cos^2\varphi\right)^{-1} = \\ &= \left(\frac{1}{D_\perp} + \left(\frac{1}{D_\parallel} - \frac{1}{D_\perp}\right)\cos^2(\varphi_{\text{M4PP}} - \varphi_0)\cos^2\phi\right)^{-1}, \end{aligned} \tag{S21}$$

such that it is given directly in the experimentally determined angles, φ_{M4PP} controlled during the M4PP measurement, and ϕ, and φ_0, which may be determined from EBSD measurements.

In the same manner, expressions for electrical conductivity and resistivity can be rewritten as

$$\begin{aligned} \sigma_{\text{eff}} &= \sqrt{\sigma_\perp\sigma_\parallel + \left(\sigma_\perp^2 - \sigma_\perp\sigma_\parallel\right)\cos^2\varphi} = \\ &= \sqrt{\sigma_\perp\sigma_\parallel + \left(\sigma_\perp^2 - \sigma_\perp\sigma_\parallel\right)\cos^2(\varphi_{\text{M4PP}} - \varphi_0)\cos^2\phi} \end{aligned} \tag{S22}$$

$$\begin{aligned} \rho_{\text{eff}} &= \left(\sqrt{\frac{1}{\rho_\perp\rho_\parallel} + \left(\frac{1}{\rho_\perp^2} - \frac{1}{\rho_\perp\rho_\parallel}\right)\cos^2\varphi}\right)^{-1} = \\ &= \left(\sqrt{\frac{1}{\rho_\perp\rho_\parallel} + \left(\frac{1}{\rho_\perp^2} - \frac{1}{\rho_\perp\rho_\parallel}\right)\cos^2(\varphi_{\text{M4PP}} - \varphi_0)\cos^2\phi}\right)^{-1}, \end{aligned} \tag{S23}$$

in terms of the angles φ_{M4PP}, φ_0 and ϕ.

The electronic contribution to thermal conductivity is

$$\kappa_e = \mathcal{L}T\sigma = \mathcal{L}T\left(\frac{\sin^2\varphi}{\sigma_\perp} + \frac{\cos^2\varphi}{\sigma_\parallel}\right)^{-1},$$

where $\mathcal{L}$ is the Lorentz number and T the absolute temperature. By use of Eq. S18 it is fairly easy to show that

$$\kappa_e = \mathcal{L}T\sigma = \frac{\mathcal{L}T}{\rho} = \mathcal{L}T\frac{\sigma_\perp^2\sigma_\parallel}{\sigma_{\text{eff}}^2} = \mathcal{L}T\frac{\rho_{\text{eff}}^2}{\rho_\perp^2\rho_\parallel}. \tag{S24}$$

This simple relation makes it possible to add meaningful experimental points to a κ_e curve where the principal conductivities or resistivities are obtained from a fit to a full angle scan, while σ_{eff} or ρ_{eff} is measured at the actual angle to the c-axis. Eq. S24 also illustrates the simple relation at any angle φ between the resistivity ρ or conductivity σ in the direction defined by the angle φ and the measured M4PP resistivity ρ_{eff} or conductivity σ_{eff} , i.e.,

$$\sigma = \frac{\sigma_\perp^2 \sigma_\parallel}{\sigma_{\text{eff}}^2} \text{ and } \rho = \frac{\rho_\perp^2 \rho_\parallel}{\rho_{\text{eff}}^2}. \tag{S25}$$

2 Methods and Materials

2.1 Crystal growth

High purity (99.999% of Alpha Aldrich) granules of Te and Bi or Sb were used in stoichiometric ratios of Bi_2Te_3 and Sb_2Te_3. 20 g of the granules were mixed in appropriate molar ratios as shown in Table S1. The weighted mixture was loaded into a quartz ampoule and sealed under the 10^{-4} Torr pressure.

x	M_w^{tot}[g/mol]	n[mol]	m_{Bi}[g]	m_{Sb}[g]	m_{Te}[g]
0	800.76	0.024976	10.4391	0	9.5609
1	623.32	0.031932	0	7.7762	12.2238

Table S1: Elemental composition. Elemental composition of $Bi_{1-x}Sb_xTe_3$ for crystal growth.

Crystals were grown by melting using a tube furnace (VECSTAR), where a uniform temperature was applied to the sealed quartz ampoule. The furnace was heated up to 980°C at a 55°C/h rate for approximately 500 minutes. Finally, the ampoule was cooled to room temperature at 55°C/h. After the growth process, the big ingot, with a diameter of 12.7 mm, was sliced into small pellets with a 2.5- 3 mm thickness using a diamond saw. The outcome of the growth process was poly-crystalline Bi_2Te_3 and Sb_2Te_3 with a large grains size of 10-300 μm. The samples were characterized using X-ray diffraction analysis (XRD), energy dispersive X-ray spectroscopy (EDS), and scanning electron microscope (SEM) are used for corresponding structure and composition confirmation. The crystal structure of both samples is a rhombohedral crystal phase (*R-3m* space group) layered along the c-axis bonded by weak van der Waals bonds shown in Fig. S4.

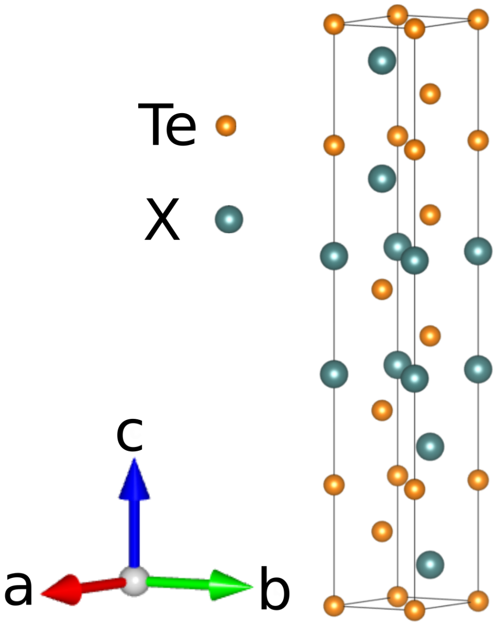

Fig. S4: Unit cell. Crystal structure of X_2Te_3 (X = Bi, Sb) plotted using VESTA, both samples having rhombohedral (*R-3m*) crystal structure, however, having slightly different bond lengths, i.e., Bi_2Te_3 (a,b = 4.386Å, c = 30.497Å) and Sb_2Te_3 (a,b = 4.264Å, c= 30.458Å).

2.2 XRD and EDS analysis

To verify the grown phase of the sample, we performed XRD on 56 μm sieved powder of Bi_2Te_3. XRD was performed using a *Rigaku Miniflex II* X-ray diffractometer with an angular range of $20° < 2\theta < 80°$, 0.01° resolution, and a scanning rate of 0.2°/min. XRD analysis was done using *Match* software, and peaks of XRD patterns were labelled with corresponding crystal planes at room temperature, illustrated in Fig. S5.

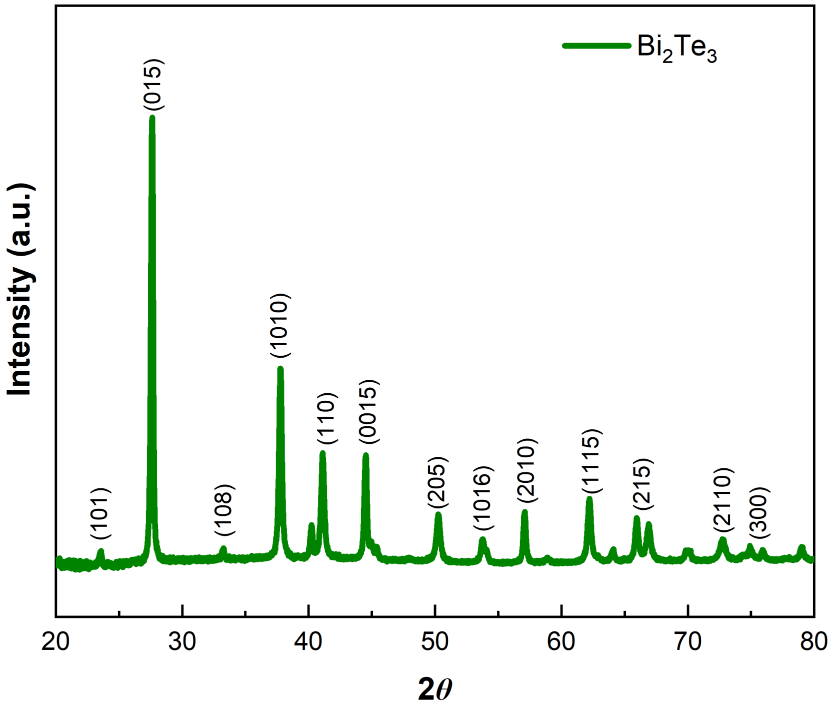

Fig. S5: Powder XRD of Bi_2Te_3. Intensity vs 2θ plot of XRD pattern collected from as-grown Bi_2Te_3 with peak labeled for corresponding planes.

Surface imaging and elemental analysis were performed using a *Zeiss Ultra* Plus high-resolution scanning electron microscope (SEM) operated at 4 kV acceleration voltage equipped with an energy dispersive X-ray spectroscopy (EDS) detector (Oxford SDD EDS detector) operated at 8 kV. Crystallographic analysis was performed using an Electron Back-Scattered Diffraction (EBSD) detector (Oxford c-nano) operated at 20 kV.

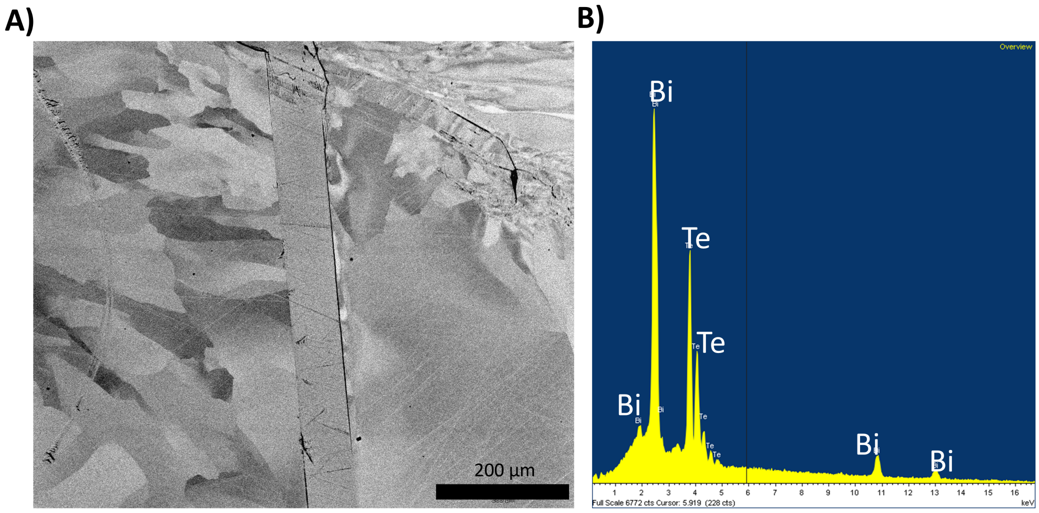

Fig. S6: Surface morphology and chemical composition. A) SEM image of Bi_2Te_3 sample. B) Peaks of elemental compositions of the location shown in Fig. S6A measured using EDS.

2.3 Laser Flash Analysis (LFA) for thermal diffusivity measurements

Reference thermal diffusivity measurements were carried out using the LFA, MicroFlash LFA-457 system (Netzsch GmbH, Selb), having instrument accuracy of ±3%. Each sample was measured perpendicular to the cut direction five times for the same conditions at RT in the air environment. The thermal diffusivity was measured in the direction of ingot growth, i.e., perpendicular to the cut direction. Before the measurement, the diameter and thickness of the ingots were measured using a vernier caliper. All the dimension measurements were repeated five times along different directions. Fig. S7 elucidate the bulk reference characterization of thermal diffusivity for both $Bi_2Te_3$and Sb_2Te_3 at 300K and agrees with previously reported values(*43,70,71*).

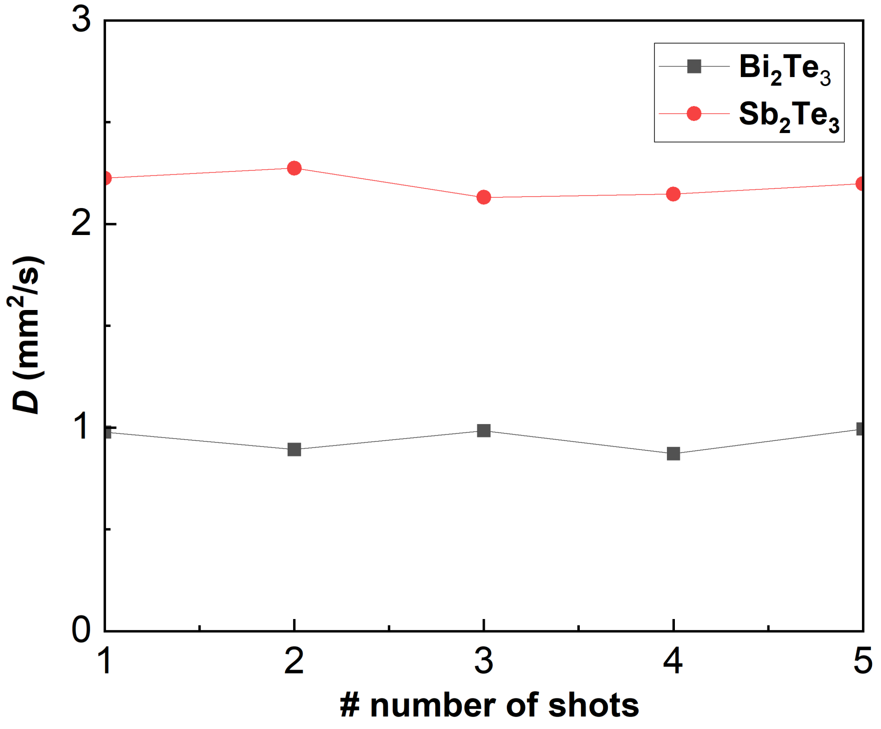

Fig. S7: Laser flash thermal diffusivity. Room temperature thermal diffusivity of Bi_2Te_3 and Sb_2Te_3 measured five times at the same conditions using LFA.

3 Area maps and line scans

The measured argument (in radian) of the Eq. S10 is shown below for M4PPs with different pitches, and the data are later used to extract thermal diffusivity. The different contrast between the 10 (Fig. S8), 20 (Fig. S9), and 30 μm (Fig. S10) pitch phases are due to the increase in the phase delay at increasing pitch. At about the boundary of larger grains for each pitch, there is high two-point load resistance near this boundary, indicating an electrically resistive boundary.

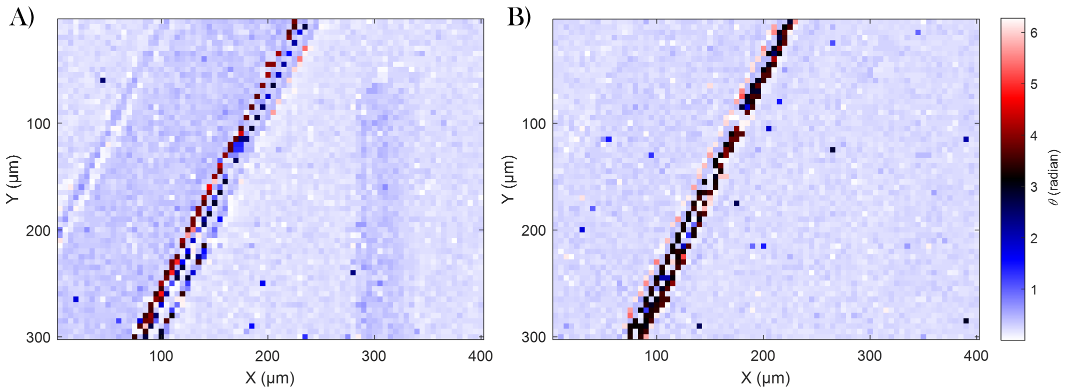

Fig. S8: Measured phase delay for 10 μm pitch. Measured phase delay corresponding to Eq. S10 for site 1 of Bi_2Te_3 for two probe orientations A) M4PP || X and B) M4PP || Y, at 10 μm probe pitch.

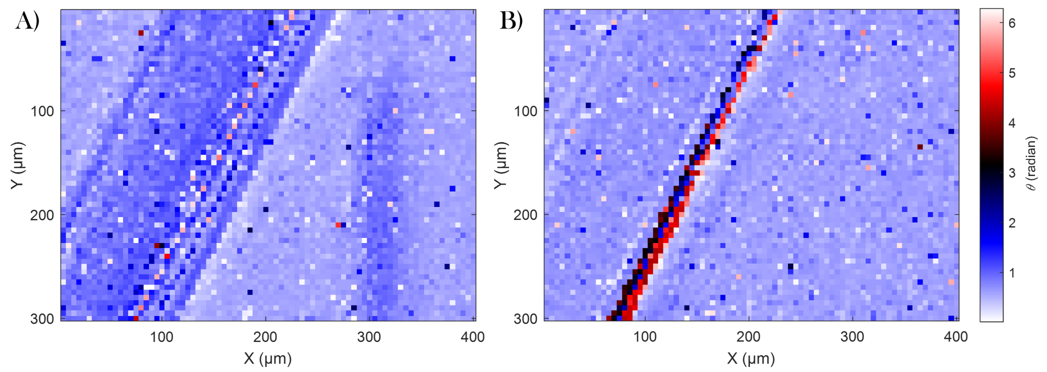

Fig. S9: Measured phase delay for 20 μm pitch. Measured phase delay corresponding to Eq. S10 for site 1 of Bi_2Te_3 for two probe orientations A) M4PP || X and B) M4PP || Y, at 20 μm probe pitch.

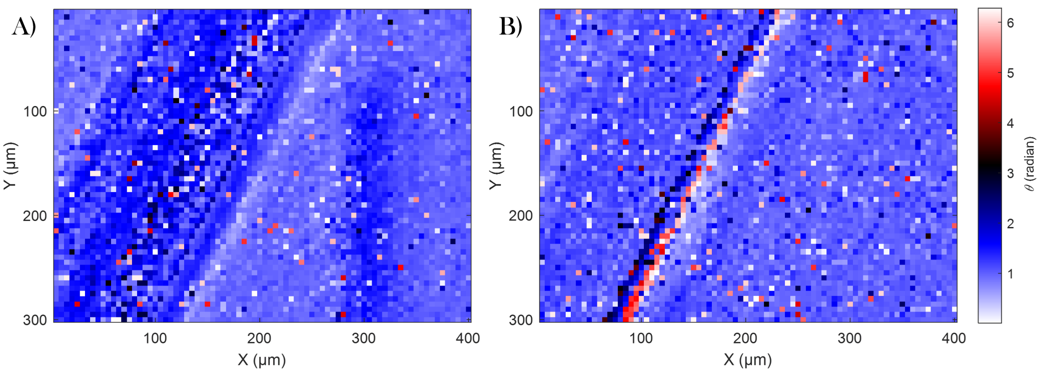

Fig. S10: Area scan of phase delay for 30 μm pitch. Measured phase delay corresponding to Eq. S10 for site 1 of Bi_2Te_3 for two probe orientations A) M4PP || X and B) M4PP || Y, at 30 μm probe pitch.

Area maps for 10 μm, 20 μm, and 30 μm probe pitch from M4PP thermal diffusivity measurement at 300K corresponding to Fig. 1 main text.

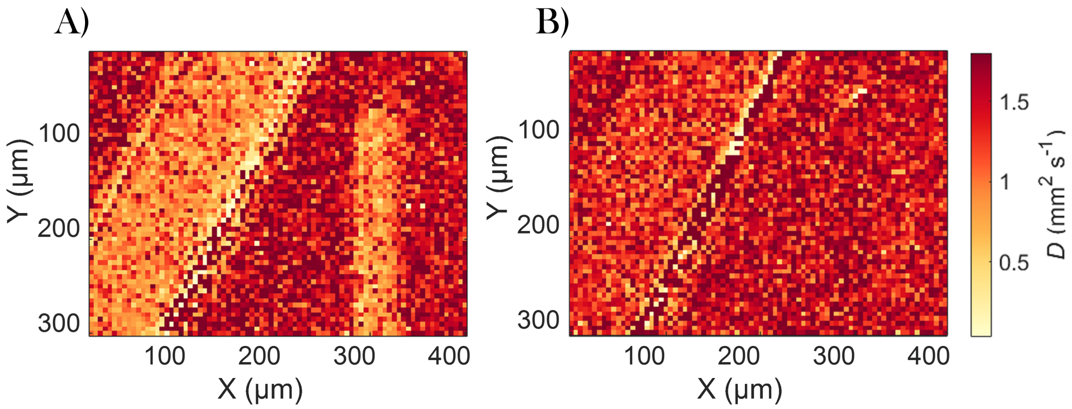

Fig. S11: Measured thermal diffusivity for 10 μm pitch. Thermal diffusivity scan for site 1 of Bi_2Te_3 at two probe orientations A) M4PP||X and B) M4PP||Y, at 10 μm probe pitch.

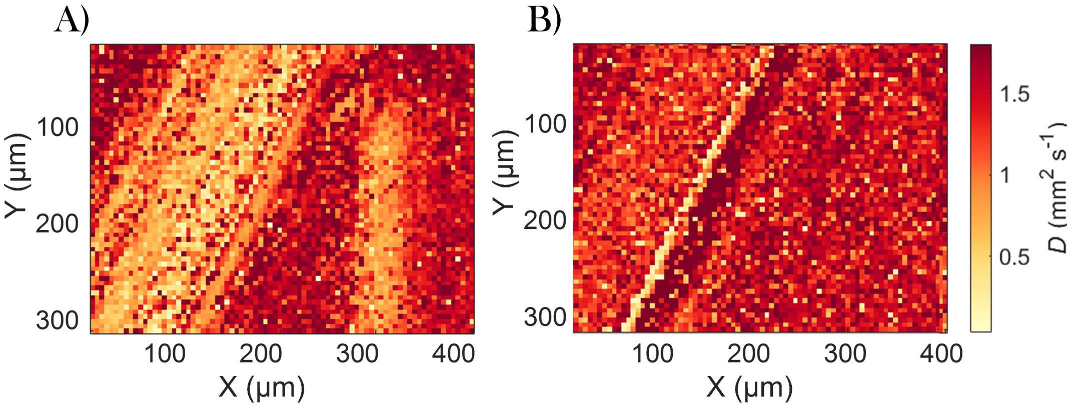

Fig. S12: Area scan of thermal diffusivity for 20 μm pitch. Thermal diffusivity scan for site 1 of Bi_2Te_3 at two probe orientations A) M4PP||X and B) M4PP||Y, at 20 μm probe pitch.

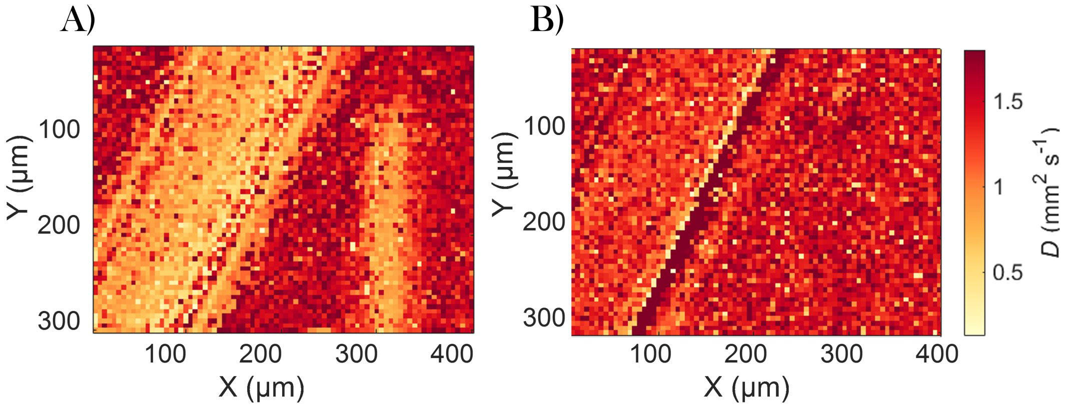

Fig. S13: Area scan of thermal diffusivity for 30 μm pitch. Thermal diffusivity scan for site 1 of Bi_2Te_3 at two probe orientations A) M4PP||X and B) M4PP||Y, at 30 μm probe pitch.

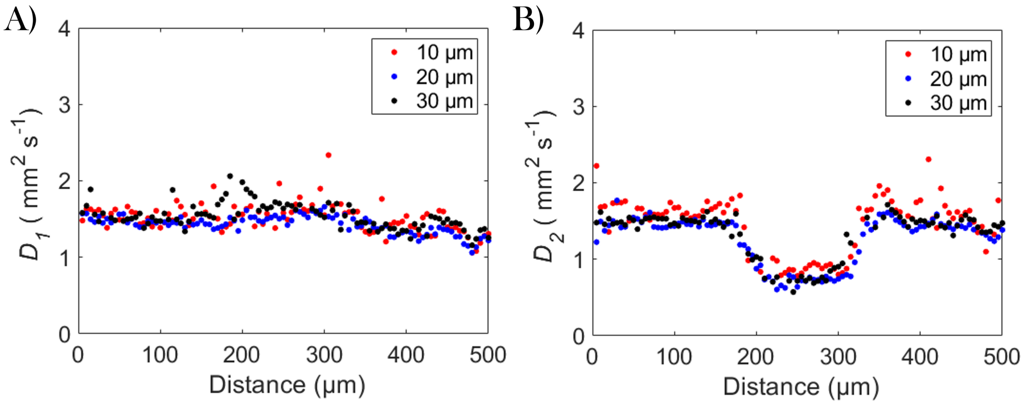

Fig. S14: Bi_2Te_3 line scans of thermal diffusivity for various pitches. Thermal diffusivity of Bi_2Te_3 measured as a line scan corresponding to Fig. 2 in the main text with two probe orientations A) M4PP||Y and B) M4PP||X, at 10, 20 and 30 μm probe pitch, respectively.

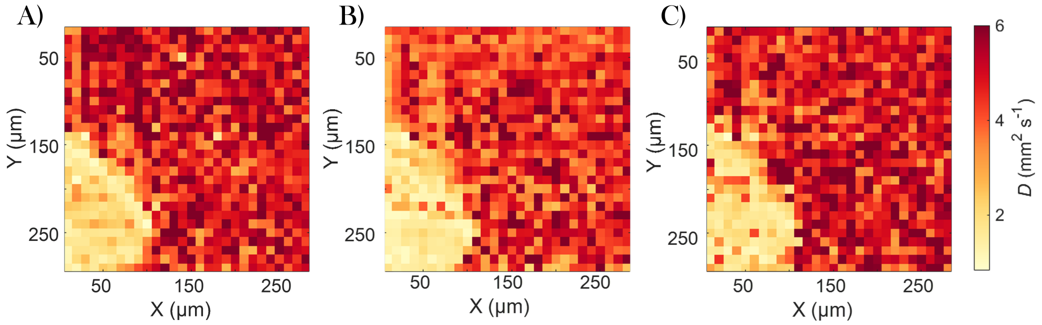

Fig. S15: Area maps of Sb_2Te_3 thermal diffusivity for various pitches. Thermal diffusivity map of Sb_2Te_3 with probe orientation M4PP||Y, at A) 10 μm, B) 20 μm and C) 30 μm probe pitch, respectively. Optical and EBSD images corresponding to the scan site are shown in Fig. S18.

4 Miller indices and resistivity from angle measurements

The thermal diffusivity measurements referred to in Fig. 3 of the main text for Site 1 and 2 confirm our sensitivity to in and out-of-plane thermal diffusivity. Table S2 highlights the Miller indices for all scan angles for both sites.

M4PP angle (°) with X	site 1,[hkl]	site 2, [hkl]
0	[$\bar{2}$ $\bar{10}$ 5]	[$\bar{2}$ $\bar{1}$ 0]
10	[2 $\bar{3}$ 2]	[$\bar{5}$ $\bar{3}$ 0]
20	[5 $\bar{2}$ 2]	[$\bar{4}$ $\bar{3}$ 0]
30	[9 $\bar{1}$ 2]	[$\bar{10}$ $\bar{9}$ 0]
40	[6 0 1]	[$\bar{8}$ $\bar{9}$ 0]
50	[9 1 1]	[$\bar{5}$ $\bar{7}$ 0]
60	[5 1 0]	[$\bar{4}$ $\bar{7}$ 0]
70	[4 1 0]	[$\bar{2}$ $\bar{5}$ 0]
80	[7 2 0]	[$\bar{1}$ $\bar{4}$ 0]
90	[3 1 0]	[0 $\bar{1}$ 0]

Table S2: Crystallographic direction. Miller indices of the crystallographic direction along the M4PP axis, calculated from EBSD data for sites 1 and 2 (cf. Fig. 3a main text).

1st harmonic M4PP resistance was used to extract the resistivity of site 1 (cf. main text Fig. 3) using Eq. S19. The measured electrical resistivity from M4PP measurement is plotted in Fig. S16 (blue filled circle). The black solid line corresponds to the fit of Eq. S18 to the measured resistivity. This procedure provides the tensor element for electrical conductivity summarized in Table S3. In this study, the effect of Peltier heating on resistivity has not been taken into account.

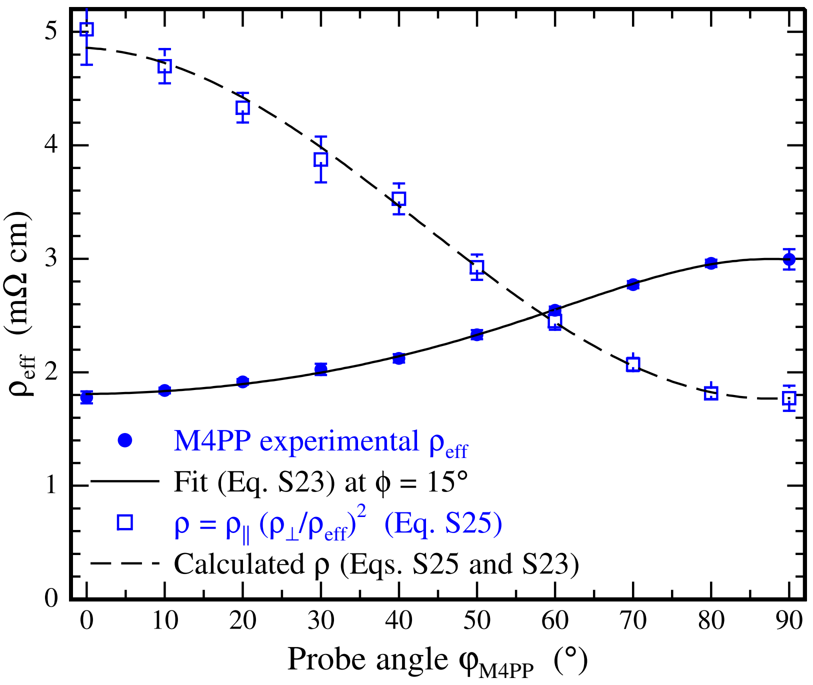

Fig. S16: Measured resistivity of Bi_2Te_3 for site 1. The filled blue circles show M4PP resistivity ρ_{eff} as function of angle φ_{M4PP} as measured on Site 1 (cf. Fig. 3a, main text) of Bi_2Te_3 using 10 μm probe pitch. The error bars are the standard deviations of 10 measurements at each angle. The full black line shows a fit of Eq. S23 to the measured ρ_{eff} data using $\phi = 15^\circ$ as determined by EBSD. The open blue squares show Bi_2Te_3 resistivity ρ as function of angle φ_{M4PP} calculated using Eq. S25 from the measured ρ_{eff} and the extracted resistivity tensor elements, $\rho_\perp$ and $\rho_\parallel$. The error bars are calculated using the usual error propagation rule. The dashed black line shows ρ calculated from the extracted resistivity tensor elements.

From the angle scanned M4PP measurements at site 1 of the main paper, the thermal diffusivity ($D_\perp$ and $D_\parallel$) and the electrical conductivity ($\sigma_\perp/\sigma_\parallel$) or resistivity ($\rho_\perp$ and $\rho_\parallel$) tensor elements for Bi_2Te_3 were extracted using Eqs. S6 and S18. From these experimental data, the thermal conductivity tensor elements ($\kappa_\perp$ and $\kappa_\parallel$) were estimated using the mass density $\varrho = 7.6\,\mathrm{g\,cm^{-3}}$, and specific heat capacity $c = 165\,\mathrm{J\,kg^{-1}\,K^{-1}}$(*63*) as $\kappa_\perp = D_\perp \varrho c$ and $\kappa_\parallel = D_\parallel \varrho c$. The electronic thermal conductivity tensor elements ($\kappa_{e\perp}$ and $\kappa_{e\parallel}$) were estimated from the electrical conductivity tensor using the Wiedemann-Franz relation ($\kappa_e/(\sigma T) = \mathcal{L}$) using $\mathcal{L} = \frac{\pi^2}{3}\left(\frac{k_{\mathrm{B}}}{\mathrm{e}}\right)^2$ where k_{B} is Boltzmann's constant and e the unit charge and T the absolute temperature. Note, this value of the Lorenz number $\mathcal{L}$ may not perfectly accurate as Bi_2Te_3 is a semiconductor(*44,67*), where the prefactor in $\mathcal{L}$ may vary from 2 to 3 for non-degenerately doped material depending on the scattering mechanism, while the prefactor $\pi^2/3$ is valid for metals and degenerately doped

semiconductors. We measure a rather high conductivity and therefore use the prefactor $\pi^2/3$. Thus, we calculate the estimated tensor elements $\kappa_{e\perp} = \sigma_\perp T\mathcal{L}$ and $\kappa_{e\parallel} = \sigma_\parallel T\mathcal{L}$ at $T = 300\,\mathrm{K}$, while the lattice thermal conductivity elements $\kappa_{p\perp}$ and $\kappa_{p\parallel}$ due to phonon transport may be estimated from $\kappa_{p\perp} = \kappa_\perp - \kappa_{e\perp}$ and $\kappa_{p\parallel} = \kappa_\parallel - \kappa_{e\parallel}$. The resulting data are summarized in Table S3. We note that the extracted resistivity tensor elements are $\rho_\perp = 1.76\,\mathrm{m\Omega\,cm}$ and $\rho_\parallel = 5.07\,\mathrm{m\Omega\,cm}$.

Symbol	D	σ	κ	κ_e	κ_p
Unit	$\mathrm{mm^2\,s^{-1}}$	$\mathrm{S\,cm^{-1}}$	$\mathrm{W\,m^{-1}\,K^{-1}}$	$\mathrm{W\,m^{-1}\,K^{-1}}$	$\mathrm{W\,m^{-1}\,K^{-1}}$
Perpendicular $\perp$	1.66	567	2.08	0.415	1.67
Parallel $\parallel$	0.74	197	0.93	0.144	0.76
Ratio $\perp/\parallel$	2.24	2.8	2.2	2.8	2.19

Table S3: Various thermal/electrical conductivity contributions. Extracted experimental tensor elements of the thermal diffusivity and electrical conductivity and derived tensor elements for thermal conductivity from site 1. The net thermal conductivity κ, the electronic thermal conductivity κ_e, and the lattice thermal conductivity κ_p tensor elements are listed. The perpendicular to parallel tensor element ratio is also listed in the last row.

Achieving independent control of thermoelectric materials' electrical and thermal conductivity remains one of the most significant challenges in improving their efficiency(*72*). Here, we present a possibility to increase the electrical to thermal conductivity ratio (σ/κ) or $\mathcal{L}T\sigma/\kappa$ (Fig. S17) by measuring it in different crystallographic directions. This type of measurement is not trivial for various classes of materials, therefore opening a pathway to test and screen the best possible thermoelectric material.

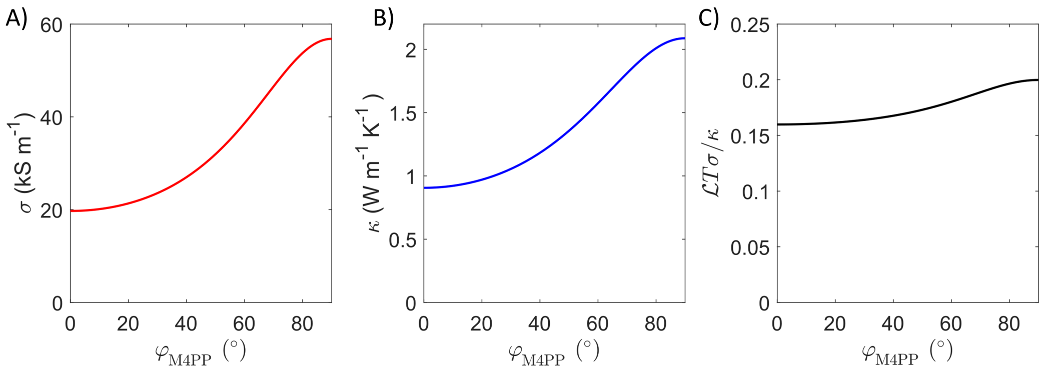

Fig. S17: Components of Wiedemann-Franz relation. A) The solid line shows the σ calculated from the tensor element of it (cf. Table S3) using Eq. S18. B) Similarly, calculated total thermal conductivity for different angles using Eq. S6 and tensor elements. C) Ratio of σ to κ multiplied by $\mathcal{L}T$ for different angles, indicating a peak around 90 degrees for 10 μm probe pitch.

Table S4 below summarizes the different reference values of the thermal conductivity of stoichiometric Bi_2Te_3.

κ ($\mathrm{W\,m^{-1}K^{-1}}$)	*(49)*	*(51)*	*(54)*
Perpendicular $\perp$	1.9	1.7	2.02
Parallel $\|$	0.85	0.77	1
Ratio ($\perp/\|$)	2.23	2.20	2.02

Table S4: Comparison of literature thermal conductivity. Reference values of thermal conductivity for Bi_2Te_3.

An overview of existing thermal diffusivity or thermal conductivity measurement techniques is summarized in Table S5.

Method	Measurement Time	Geometry	Need Calibration	Sample Preparation	Resolves anisotropy?	Disadvantage	Advantage
Laser flash	1-2 s	Ø<25 mm<br>Plane parallel	Yes	Absorber coating	No	Only macroscopic characterization, sample must have good emission and absorption capability.	Wide temperature range, fast, accurate at high temperatures.
3ω-method	1-2 s	Device with electrodes	Yes	Complicated	No	Complicated fitting procedure and device fabrication.	Wide temperature range, accurate Insensitive to black-body radiation.
TDTR, FDTR, SDTR, TR-MOKE	10-20 ns	Flat bulk and thin film sample in µm thickness.	Yes	Metal coating	Yes	Coating a thin metal layer on the sample and complicated fitting procedures.	Wide temperature range, accurate and fast.
M4PP (this result)	1-10 s	Ø>0.25 mm<br>Ø<300 mm<br>Flat surface	No	None	Yes	Can not measure on insulating material. Currently limited to 300K.	Calibration-free, insensitive to sample surface and its optical properties. Fast and accurate. Free from complicated fitting procedures and additional sample preparation. It can measure accurately on both isotropic and anisotropic on same samples with <3% precision.

Table S5: Comparison of various techniques. Overview of the existing and recent state-of-the-art thermal diffusivity measurement techniques (*23,28,49,66*).

5 Electron Back-Scattered Diffraction (EBSD)

EBSD measures the crystallographic orientation of individual grains and grain size distribution. Polishing is a pivotal preparation step for imaging the grains using EBSD—the surface of the crystal has to be uniform to show the diffraction patterns. To polish the samples (for EBSD), the samples were mounted on a stable horizontal stage, i.e., a plastic holder. After polishing, the samples were argon (Ar) ion milled. EBSD patterns were recorded on both Bi_2Te_3 and Sb_2Te_3 samples, where three interesting sites were discovered and later used for M4PP measurement. EBSD sites for Bi_2Te_3 are already shown in the main text; below we show EBSD corresponding to Sb_2Te_3.

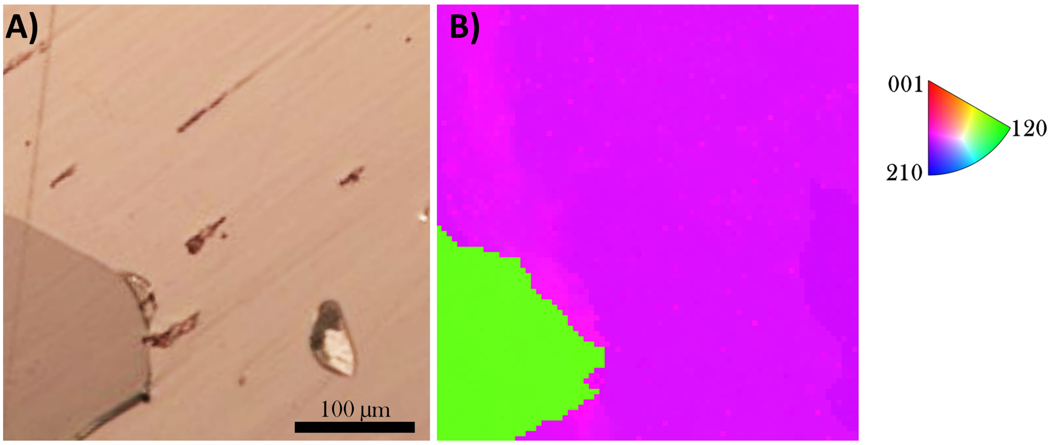

Fig. S18: Surface morphology. A) Polarised optical microscope image and B) Inverse pole figure (IPF) map colored based on the z-direction for Sb_2Te_3.

6 Focused ion beam lamella

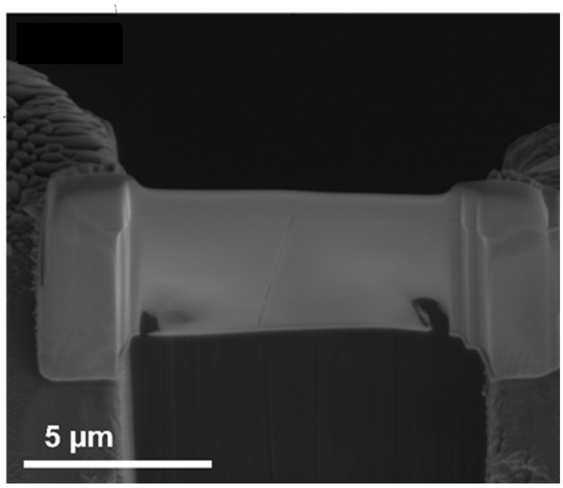

Fig. S19: Lamella from HAADF-STEM. Secondary electron image of prepared FIB lamella used for the STEM measurement of Bi_2Te_3 corresponding to the HAADF-STEM image shown in Fig. 3 main text.

We employ scanning transmission electron microscopy (STEM) to obtain high-resolution images of materials at the atomic scale. To prepare our specimens for this process, we utilize a focused ion beam (FIB) to carve out thin lamella sections see Fig. S19. STEM allowed us to obtain the detailed crystallographic directions of our materials at the atomic level.

REFERENCES AND NOTES

1. K. Biswas, J. He, I. D. Blum, C.-I. Wu, T. P. Hogan, D. N. Seidman, V. P. Dravid, M. G. Kanatzidis, High-performance bulk thermoelectrics with all-scale hierarchical architectures. *Nature* **489**, 414–418 (2012).

2. Y. Yin, K. Baskaran, A. Tiwari, A review of strategies for developing promising thermoelectric materials by controlling thermal conduction. *Physica Status Solidi* **216**, 1800904 (2019).

3. L.-D. Zhao, S.-H. Lo, Y. Zhang, H. Sun, G. Tan, C. Uher, C. Wolverton, V. P. Dravid, M. G. Kanatzidis, Ultralow thermal conductivity and high thermoelectric figure of merit in SnSe crystals. *Nature* **508**, 373–377 (2014).

4. J. He, T. M. Tritt, Advances in thermoelectric materials research: Looking back and moving forward. *Science* **357**, eaak9997 (2017).

5. B. Poudel, Q. Hao, Y. Ma, Y. Lan, A. Minnich, B. Yu, X. Yan, D. Wang, A. Muto, D. Vashaee, X. Chen, J. Liu, M. S. Dresselhaus, G. Chen, Z. Ren, High-thermoelectric performance of nanostructured bismuth antimony telluride bulk alloys. *Science* **320**, 634–638 (2008).

6. A. Zevalkink, D. M. Smiadak, J. L. Blackburn, A. J. Ferguson, M. L. Chabinyc, O. Delaire, J. Wang, K. Kovnir, J. Martin, L. T. Schelhas, T. D. Sparks, S. D. Kang, M. T. Dylla, G. J. Snyder, B. R. Ortiz, E. S. Toberer, A practical field guide to thermoelectrics: Fundamentals, synthesis, and characterization. *Appl. Phys. Rev.* **5**, 021303 (2018).

7. L. Fan, J. M. Khodadadi, Thermal conductivity enhancement of phase change materials for thermal energy storage: A review. *Renew. Sustain. Energy Rev.* **15**, 24–46 (2011).

8. X. Zhang, X. Zhuo, Z. Fan, J. Mao, C. Deng, C. Deng, X. Mei, J. Cui, K. Zhou, M. Liu, Al2O3-modified 7YSZ thermal barrier coatings for protection against volcanic ash corrosion. *npj Materials Degradation* **6**, 89 (2022).

9. N. P. Padture, M. Gell, E. H. Jordan, Thermal barrier coatings for gas-turbine engine applications. *Science* **296**, 280–284 (2002).

10. F. Hu, Z.-P. Xie, J. Zhang, Z.-L. Hu, D. An, Promising high-thermal-conductivity substrate material for high-power electronic device: Silicon nitride ceramics. *Rare Metals* **39**, 463–478 (2020).

11. A. Palacios, L. Cong, M. E. Navarro, Y. Ding, C. Barreneche, Thermal conductivity measurement techniques for characterizing thermal energy storage materials–A review. *Renewable and Sustainable Energy Reviews* **108**, 32–52 (2019).

12. A. Gowda, A. Zhong, D. Esler, J. David, T. Sandeep, K. Srihari, F. Schattenmann, Design of a high reliability and low thermal resistance interface material for microelectronics, in *Proceedings of the 5th Electronics Packaging Technology Conference (EPTC 2003)* (IEEE, 2003), pp. 557–562.

13. J. Due, A. J. Robinson, Reliability of thermal interface materials: A review. *Appl. Therm. Eng.* **50**, 455–463 (2013).

14. S. E. Kim, F. Mujid, A. Rai, F. Eriksson, J. Suh, P. Poddar, A. Ray, C. Park, E. Fransson, Y. Zhong, D. A. Muller, P. Erhart, D. G. Cahill, J. Park, Extremely anisotropic van der Waals thermal conductors. *Nature* **597**, 660–665 (2021).

15. A. J. Minnich, Exploring the extremes of heat conduction in anisotropic materials. *Microscale thermophysical engineering* **20**, 1–21 (2016).

16. A. B. Robbins, S. X. Drakopoulos, I. Martin-Fabiani, S. Ronca, A. J. Minnich, Ballistic thermal phonons traversing nanocrystalline domains in oriented polyethylene. *Proc. Natl. Acad. Sci.* **116**, 17163–17168 (2019).

17. F. Sun, S. Mishra, P. H. McGuinness, Z. H. Filipiak, I. Marković, D. A. Sokolov, N. Kikugawa, J. W. Orenstein, S. A. Hartnoll, A. P. Mackenzie, V. Sunko, A spatially resolved optical method to measure thermal diffusivity. *Rev. Sci. Instrum.* **94**, 043003 (2023).

18. H. Dong, B. Wen, R. Melnik, Relative importance of grain boundaries and size effects in thermal conductivity of nanocrystalline materials. *Sci. Rep.* **4**, 7037 (2014).

19. G. Dehm, J. Cairney, Implication of grain-boundary structure and chemistry on plasticity and failure. *MRS Bulletin* **47**, 800–807 (2022).

20. H. Bishara, M. Ghidelli, G. Dehm, Approaches to measure the resistivity of grain boundaries in metals with high sensitivity and spatial resolution: A case study employing Cu. *ACS Applied Electronic Materials* **2**, 2049–2056 (2020).

21. H. Bishara, S. Lee, T. Brink, M. Ghidelli, G. Dehm, Understanding grain boundary electrical resistivity in Cu: The effect of boundary structure. *ACS Nano* **15**, 16607–16615 (2021).

22. E. Isotta, S. Jiang, G. Moller, A. Zevalkink, G. J. Snyder, O. Balogun, Microscale imaging of thermal conductivity suppression at grain boundaries. *Adv. Mater.*, **35**, 2302777 (2023).

23. W. J. Parker, R. J. Jenkins, C. P. Butler, G. L. Abbott, Flash method of determining thermal diffusivity, heat capacity, and thermal conductivity. *J. Appl. Phys.* **32**, 1679–1684 (1961).

24. X. Yan, B. Poudel, Y. Ma, W. S. Liu, G. Joshi, H. Wang, Y. Lan, D. Wang, G. Chen, Z. F. Ren, Experimental studies on anisotropic thermoelectric properties and structures of n-type $Bi_2Te_{2.7}Se_{0.3}$. *Nano Lett.* **10**, 3373–3378 (2010).

25. B. L. Adams, S. I. Wright, K. Kunze, Orientation imaging: The emergence of a new microscopy. *Metall. Trans. A.* **24**, 819–831 (1993).

26. G. L. Burton, S. Wright, A. Stokes, D. R. Diercks, A. Clarke, B. P. Gorman, Orientation mapping with Kikuchi patterns generated from a focused STEM probe and indexing with commercially available EDAX software. *Ultramicroscopy* **209**, 112882 (2020).

27. C. Gayner, Y. Natanzon, Y. Kauffmann, Y. Amouyal, Topologically-enhanced thermoelectric properties in Bi2Te3-based compounds: Effects of grain size and misorientation. *ACS Appl. Mater. Interfaces* **14**, 49730–49745 (2022).

28. V. Mishra, C. L. Hardin, J. E. Garay, C. Dames, A 3 omega method to measure an arbitrary anisotropic thermal conductivity tensor. *Review of scientific instruments* **86**, 054902 (2015).

29. P. Jiang, X. Qian, R. Yang, Time-domain thermoreflectance (TDTR) measurements of anisotropic thermal conductivity using a variable spot size approach. *Rev. Sci. Instrum.* **88**, 074901 (2017).

30. D. H. Olson, J. L. Braun, P. E. Hopkins, Spatially resolved thermoreflectance techniques for thermal conductivity measurements from the nanoscale to the mesoscale. *J. Appl. Phys.* **126**, 150901 (2019).

31. D. G. Cahill, Thermal-conductivity measurement by time-domain thermoreflectance. *MRS Bulletin* **43**, 782–789 (2018).

32. J. Yang, C. Maragliano, A. J. Schmidt, Thermal property microscopy with frequency domain thermoreflectance. *Review of Scientific Instruments* **84** (2013).

33. P. Jiang, D. Wang, Z. Xiang, R. Yang, H. Ban, A new spatial-domain thermoreflectance method to measure a broad range of anisotropic in-plane thermal conductivity. *International Journal of Heat and Mass Transfer* **191**, 122849 (2022).

34. R. J. Warzoha, N. T. Vu, B. F. Donovan, E. Cimpoiasu, D. J. Sharar, A. C. Leff, A. A. Wilson, A. N. Smith, Grain growth-induced thermal property enhancement of NiTi shape memory alloys for elastocaloric refrigeration and thermal energy storage systems. *Int. J. Heat Mass Transfer* **154**, 119760 (2020).

35. J. P. Feser, J. Liu, D. G. Cahill, Pump-probe measurements of the thermal conductivity tensor for materials lacking in-plane symmetry. *Rev. Sci. Instrum.* **85,** 104903 (2014).

36. J. Liu, G.-M. Choi, D. G. Cahill, Measurement of the anisotropic thermal conductivity of molybdenum disulfide by the time-resolved magneto-optic Kerr effect. *J. Appl. Phys.* **116,** 233107 (2014).

37. J. Zhu, H. Park, J.-Y. Chen, X. Gu, H. Zhang, S. Karthikeyan, N. Wendel, S. A. Campbell, M. Dawber, X. Du, M. Li, J.-P. Wang, R. Yang, X. Wang, Revealing the origins of 3D anisotropic thermal conductivities of black phosphorus. *Adv. Electron. Mater.* **2**, 1600040 (2016).

38. D. Davydova, A. de la Cadena, D. Akimov, B. Dietzek, Transient absorption microscopy: Advances in chemical imaging of photoinduced dynamics. *Laser Photonics Rev.* **10**, 62–81 (2016).

39. T. Luo, G. Chen, Nanoscale heat transfer – from computation to experiment. *Phys. Chem. Chem. Phys.* **15**, 3389–3412 (2013).

40. S. Thorsteinsson, F. Wang, D. H. Petersen, T. M. Hansen, D. Kjær, R. Lin, J.-Y. Kim, P. F. Nielsen, O. Hansen, Accurate microfour-point probe sheet resistance measurements on small samples. *Rev. Sci. Instrum.* **80**, 053902 (2009).

41. D. H. Petersen, O. Hansen, T. M. Hansen, P. Bøggild, R. Lin, D. Kjær, P. F. Nielsen, T. Clarysse, W. Vandervorst, E. Rosseel, N. S. Bennett, N. E. B. Cowern, Review of electrical characterization of ultra-shallow junctions with micro four-point probes. *J. Vac. Sci. Technol. B* **28**, C1C27–C1C33 (2010).

42. D. Kjaer, R. Lin, D. H. Petersen, P. M. Kopalidis, R. Eddy, D. A. Walker, W. F. Egelhoff, L. Pickert, Micro Four-Point Probe with High Spatial Resolution for Ion Implantation and Ultra-Shallow Junction Characterization in *AIP Conference Proceedings* (AIP, 2008) vol. 1066, p. 167.

43. B. Beltrán-Pitarch, B. Guralnik, N. Lamba, A. R. Stilling-Andersen, L. Nørregaard, T. M. Hansen, O. Hansen, N. Pryds, P. F. Nielsen, D. H. Petersen, Determination of thermal diffusivity of thermoelectric materials using a micro four-point probe method. *Mater. Today Phys.* **31**, 100963 (2023).

44. C. Gayner, L. T. Menezes, Y. Natanzon, Y. Kauffmann, H. Kleinke, Y. Amouyal, Development of nanostructured Bi_2Te_3 with high thermoelectric performance by scalable synthesis and microstructure manipulations. *ACS Appl. Mater. Interfaces* **15**, 13012–13024 (2023).

45. R. J. Mehta, Y. Zhang, C. Karthik, B. Singh, R. W. Siegel, T. Borca-Tasciuc, G. Ramanath, A new class of doped nanobulk high-figure-of-merit thermoelectrics by scalable bottom-up assembly. *Nat. Mater.* **11**, 233–240 (2012).

46. S. Izadi, J. W. Han, S. Salloum, U. Wolff, L. Schnatmann, A. Asaithambi, S. Matschy, H. Schlörb, H. Reith, N. Perez, K. Nielsch, S. Schulz, M. Mittendorff, G. Schierning, Interface-dominated topological transport in nanograined Bulk Bi_2Te_3. *Small* **17**, e2103281 (2021).

47. C. Gayner, Y. Amouyal, Energy filtering of charge carriers: Current trends, challenges, and prospects for thermoelectric materials. *Adv. Funct. Mater.* **30**, 1901789 (2020).

48. G. Solomon, E. Song, C. Gayner, J. A. Martinez, Y. Amouyal, Effects of microstructure and neodymium doping on Bi_2Te_3 nanostructures: Implications for thermoelectric performance. *ACS Appl Nano Mater* **4**, 4419–4431 (2021).

49. Y. Liu, Y. Zhang, K. H. Lim, M. Ibáñez, S. Ortega, M. Li, J. David, S. Martí-Sánchez, K. M. Ng, J. Arbiol, M. V. Kovalenko, D. Cadavid, A. Cabot, High thermoelectric performance in crystallographically textured n-Type $Bi_2Te_{3-x}Se_x$Produced from asymmetric colloidal nanocrystals. *ACS Nano* **12**, 7174–7184 (2018).

50. B.-L. Huang, M. Kaviany, Ab initio and molecular dynamics predictions for electron and phonon transport in bismuth telluride. *Phys. Rev. B* **77**, 125209 (2008).

51. H. J. Goldsmid, The thermal conductivity of bismuth telluride. *Proc. Phys. Soc. B* **69**, 203–209 (1956).

52. J. P. Fleurial, L. Gailliard, R. Triboulet, H. Scherrer, S. Scherrer, Thermal properties of high quality single crystals of bismuth telluride—Part I: Experimental characterization. *J Phys Chem Solids* **49**, 1237–1247 (1988).

53. M. Carle, P. Pierrat, C. Lahalle-Gravier, S. Scherrer, H. Scherrer, Transport properties of n-type $Bi_2(Te_{1-x}Se_x)_3$ single crystal solid solutions ($x \leqslant 0.05$). *J Phys Chem Solids* **56**, 201–209 (1995).

54. D. M. Rowe, *CRC Handbook of Thermoelectrics* (CRC press, 2018).

55. D. C. Worledge, P. L. Trouilloud, Magnetoresistance measurement of unpatterned magnetic tunnel junction wafers by current-in-plane tunneling. *Appl. Phys. Lett.* **83**, 84–86 (2003).

56. B. Qiu, X. Ruan, Molecular dynamics simulations of lattice thermal conductivity of bismuth telluride using two-body interatomic potentials. *Physical Review B* **80**, 165203 (2009).

57. A. Baranovskiy, A. Graff, J. Klose, J. Mayer, Y. Amouyal, On the origin of vibrational properties of calcium manganate based thermoelectric compounds. *Nano Energy* **47**, 451–462 (2018).

58. A. Baranovskiy, M. Harush, Y. Amouyal, On the influence of rare earth dopants on thermal transport in thermoelectric Bi_2Te_3 compounds: An ab initio perspective. *Advcd Theory and Sims* **2**, 1800162 (2019).

59. Q. Shi, J. Li, X. Zhao, Y. Chen, F. Zhang, Y. Zhong, R. Ang, Comprehensive insight into *p*-type Bi_2Te_3-based thermoelectrics near room temperature. *ACS Appl. Mater. Interfaces* **14**, 49425–49445 (2022).

60. C. B. Satterthwaite, R. W. Ure Jr., Electrical and thermal properties of Bi_2 Te_3. *Phys. Rev.* **108**, 1164–1170 (1957).

61. I. Lobato, T. Friedrich, S. Van Aert, Deep convolutional neural networks to restore single-shot electron microscopy images. *npj Computational Materials* **10**, 10 (2024).

62. C. L. Petersen, R. Lin, D. H. Petersen, P. F. Nielsen, Micro-scale sheet resistance measurements on ultra shallow junctions in *2006 14th IEEE International Conference on Advanced Thermal Processing of Semiconductors* (IEEE, 2006), pp. 153–158.

63. F. Wang, D. H. Petersen, H. V. Jensen, C. Hansen, D. Mortensen, L. Friis, O. Hansen, Three-way flexible cantilever probes for static contact. *J. Micromech. Microeng.* **21**, 085003 (2011).

64. T. Clarysse, A. Moussa, F. Leys, R. Loo, W. Vandervorst, M. C. Benjamin, R. J. Hillard, V. N. Faifer, M. I. Current, R. Lin, D. H. Petersen, Accurate sheet resistance measurement on ultra-shallow profiles. *MRS Online Proceedings Library* **912**, 507 (2006).

65. N. Lamba, B. Guralnik, B. Beltrán-Pitarch, V. Rosendal, N. Pryds, O. Hansen, D. H. Petersen, Deconvolution of heat sources for application in thermoelectric micro four-point probe measurements. *Int. J. Therm. Sci.* **196**, 108716 (2024).

66. T. Arisaka, M. Otsuka, Y. Hasegawa, Measurement of thermal conductivity and specific heat by impedance spectroscopy of Bi_2Te_3 thermoelectric element. *Rev. Sci. Instrum.* **90**, 046104 (2019).

67. C. Kittel, *Introduction to solid state physics* (John Wiley & Sons Inc., 1986).

68. H. S. Carslaw, J. C. Jaeger, *Conduction of Heat in Solids* (Clarendon Press, 1959).

69. L. B. Valdes, Resistivity measurements on germanium for transistors. *Proceedings of the IRE* **42**, 420–427 (1954).

70. H.-W. Jeon, H.-P. Ha, D.-B. Hyun, J.-D. Shim, Electrical and thermoelectrical properties of undoped Bi_2Te_3-Sb_2Te_3 and Bi_2Te_3-Sb_2Te_3-Sb_2Se_3 single crystals. *J Phys Chem Solids* **52**, 579–585 (1991).

71. J. Shi, X. Chen, W. Wang, H. Chen, A new rapid synthesis of thermoelectric Sb2Te3 ingots using selective laser melting 3D printing. *Mater. Sci. Semicond. Process.* **123**, 105551 (2021).

72. W. H. Nam, Y. S. Lim, W. Kim, H. K. Seo, K. S. Dae, S. Lee, W.-S. Seo, J. Y. Lee, A gigantically increased ratio of electrical to thermal conductivity and synergistically enhanced thermoelectric properties in interface-controlled TiO 2–RGO nanocomposites. *Nanoscale* **9**, 7830–7838 (2017).
